# Immune landscape and risk prediction based on pyroptosis-related molecular subtypes in triple-negative breast cancer

**DOI:** 10.3389/fimmu.2022.933703

**Published:** 2022-09-16

**Authors:** Lixi Luo, Qun Wei, Chenpu Xu, Minjun Dong, Wenhe Zhao

**Affiliations:** Department of Surgical Oncology, Sir Run Run Shaw Hospital, Zhejiang University School of Medicine, Hangzhou, China

**Keywords:** pyroptosis, tumor microenvironment, triple-negative breast cancer, prognosis, immune checkpoints

## Abstract

The survival outcome of triple-negative breast cancer (TNBC) remains poor, with difficulties still existing in prognosis assessment and patient stratification. Pyroptosis, a newly discovered form of programmed cell death, is involved in cancer pathogenesis and progression. The role of pyroptosis in the tumor microenvironment (TME) of TNBC has not been fully elucidated. In this study, we disclosed global alterations in 58 pyroptosis-related genes at somatic mutation and transcriptional levels in TNBC samples collected from The Cancer Genome Atlas and Gene Expression Omnibus databases. Based on the expression patterns of genes related to pyroptosis, we identified two molecular subtypes that harbored different TME characteristics and survival outcomes. Then, based on differentially expressed genes between two subtypes, we established a 12-gene score with robust efficacy in predicting short- and long-term overall survival of TNBC. Patients at low risk exhibited a significantly better prognosis, more antitumor immune cell infiltration, and higher expression of immune checkpoints including PD-1, PD-L1, CTLA-4, and LAG3. The comprehensive analysis of the immune landscape in TNBC indicated that alterations in pyroptosis-related genes were closely related to the formation of the immune microenvironment and the intensity of the anticancer response. The 12-gene score provided new information on the risk stratification and immunotherapy strategy for highly heterogeneous patients with TNBC.

## Introduction

Breast cancer (BC) is the leading cause of cancer death among women. Triple-negative breast cancer (TNBC), marked by negative expression of estrogen receptor (ER), progesterone receptor (PR), and human epidermal growth factor receptor-2 (HER2), is the most challenging subtype of BC due to its high heterogeneity and lack of effective target therapies (1). Tumor mutation burden (TMB) is higher in TNBC than in other subtypes, suggesting a higher probability of benefits from treatment with immune checkpoint inhibitors (ICIs) (2). Several clinical trials have investigated the feasibility of adding ICIs to chemotherapy in TNBC, and most of these studies focus on inhibitors of the PD-1/PD-L1 pathway. Pembrolizumab, an anti-PD-1 agent, has been demonstrated to be helpful in improving the survival of both metastatic and early stage TNBCs (3, 4). Meanwhile, the anti-PD-L1 agent atezolizumab shows discrepant efficacy in advanced TNBC when combined with paclitaxel or nab-paclitaxel (5, 6). Overexpression of CTLA-4, another immune checkpoint molecule, is observed in breast tumors (7). A single-arm pilot study suggested treatment responses to combined anti-CTLA-4 antibody tremelimumab and anti-PD-L1 durvalumab in three of the total of seven TNBC cases investigated (8). However, the study was terminated due to the low overall response rate that did not meet the required criteria. Currently, randomized trials exploring anti-CTLA-4 treatment in TNBC are ongoing, while no positive results have been reported (9, 10). It is worth mentioning that, compared to other cancers, the TMB or microsatellite instability (MSI) in BC is still notably low (11, 12), resulting in a less dramatic response to immunotherapy. The narrow therapeutic window and ultimate drug resistance also remain to be problems that need to be resolved.

Pyroptosis is a cytolytic and inflammatory form of programmed cell death mediated by proteins from the gasdermin family (13). It is characterized by pore formation and cell swelling, followed by rupture of the plasma membrane and release of cytokines, which trigger inflammatory responses and cell death (14). Increasing evidence reveals various roles for pyroptosis in cancer pathogenesis and progression (15). Pyroptosis that occurs in only a fraction of tumor cells can induce robust antitumor immunity and synergizes with anti-PD-1 blockade (16). Gasdermin E (GSDME) expression is inhibited in many types of cancer, including BC, and tumor GSDME can activate pyroptosis, improving tumor suppression through killer cytotoxic lymphocytes (17). The GSDME promoter was found to be methylated in primary BC tissues with high frequency, and the GSDME methylation status could increase the risk of lymph node metastasis in BC patients (18). In contrast, gasdermin B (GSDMB) is upregulated in breast carcinoma and is correlated with increased tumor cell invasiveness and poor survival in patients (19). Anti-GSDMB antibody loaded onto nanocapsules efficiently reduces the aggressiveness of HER2-positive BC (20).

There are emerging studies suggesting the crosstalk between pyroptosis and the tumor microenvironment (TME) (21). The expression of gasdermin D (GSDMD), accompanied by the upstream components of the NLRP3 inflammasome, is related to the activation of the inflammasome in the tumor. NLRP3 signaling in macrophages drives the establishment of an immune-suppressive TME in pancreatic ductal adenocarcinoma (22). GSDME expression suppresses tumor growth by enhancing the functionality of tumor-infiltrating natural-killer (NK) and CD8+ T lymphocytes (17). Currently, few studies have elucidated the collaborative effects of combined pyroptosis-related genes (PRG) on BC pathogenesis. Given the complex roles of PRGs in cancer, a comprehensive understanding of PGR-mediated TME alterations in TNBC is needed to provide new insights into patients’ risk prediction and treatment decision.

This study disclosed the global characteristics of PRG alterations in TNBC and defined two distinct molecular subtypes based on 58 PRGs. Using differentially expressed genes (DEGs) between pyroptosis subtypes, we constructed a 12-gene-based risk score capable of predicting long-term overall survival (OS). A comprehensive characterization of the immune landscape in TNBC was performed based on the risk stratification system, paving the way for the identification of optimal candidates and potential regimens for efficient immunotherapy in TNBC.

## Materials and methods

### Data sources

**Supplementary Figure S1** shows the workflow of this study. BC samples were obtained from The Cancer Genome Atlas (TCGA) (https://portal.gdc.cancer.gov/) and Gene Expression Omnibus (GEO) (https://www.ncbi.nlm.nih.gov/geo/) databases. A total of 1,366 samples were involved in this study (including 113 normal tissues and 1,253 primary BC tumors). The transcriptome profiling of 542 TNBC samples was collected from three cohorts, including 162 samples from the TCGA-BRCA program, 273 from the GSE96058 dataset, and 107 from the GSE58812 dataset. Of these, cases from TCGA-BRCA and GSE96058 were used as the development cohort for differential analysis, risk model establishment, and internal validation, while the GSE58812 dataset acted as an independent external validation cohort. The fragments per kilobase million (FPKM) values in the RNA sequencing data were transformed into transcripts per kilobase million (TPM). Gene expression data derived from both RNA sequencing and microarray were combined, with batch effects removed using the “ComBat” function in R. Clinicopathological information was collected, including patient phenotype (age, menopausal status, stage, and tumor grade) and survival endpoints (vital status, days to the last follow-up, and days to death). Masked somatic mutation data of 144 TNBC samples and 986 all BC samples were retrieved from the TCGA-BRCA database.

### Mutation analysis of PRGs

Fifty-eight PRGs were collected from the “REACTOME_PYROPTOSIS” gene set of the GSEA/MSigDB Team (http://www.broad.mit.edu/gsea/msigdb/) and published papers (23–25), as shown in **Supplementary Table S1**. The somatic mutations of the 58 PRGs were visualized using the “maftools” and “RCircos” packages in R software.

### Identification of differentially expressed PRGs and prognostic PRGs

Package “limma” was used to identify differentially expressed PRGs between normal and TNBC samples. The correlations between PRG expression and OS of the patients were drawn using Kaplan–Meier curves using the “survival” and “survminer” packages.

### Consensus clustering of PRGs and functional enrichment

To build a classification of molecular subtypes for TNBC, consensus clustering was performed based on patient PRG expression patterns using the package “ConsensusClusterPlus.” The maximum number of clusters, K, was defined as 9 to draw the heatmaps from the consensus matrix. The optimal K was then determined from 2 to 9 according to the consensus matrix heatmap and the cumulative distribution function (CDF) curves. Principal component analysis (PCA) was performed to verify the disparity in pyroptosis transcription profiles among distinct subtypes. Differences in PRG expression levels, clinicopathological characteristics, and OS among different subtypes were compared. The expression of PD-1 and PD-L1 was also analyzed. The Kyoto Encyclopedia of Genes and Genomes (KEGG) signaling pathways in which different subtypes were involved were investigated by gene set variation analysis (GSVA) using the MSigDB curated gene set “c2.cp.kegg.v7.4.” The abundance of immune cells infiltrating the TNBC TME was assessed by single-sample gene set enrichment analysis (ssGSEA).

### Determination of DEGs among pyroptosis subtypes

DEGs among different pyroptosis subtypes were determined using a “limma” package with a log2-fold change > 1.5 and an adjusted p-value < 0.05. Functional enrichment of these subtype-related DEGs was performed using Gene Ontology (GO) annotation and KEGG pathway analysis with the “clusterProfiler” package.

### Development of a DEG-based risk score and determination of prognostic predictors

Univariate Cox regression was conducted to identify OS-related genes from subtype-based DEGs. Consensus clustering, according to the expression of these prognostic DEGs, was conducted to categorize tumors into distinct gene subtypes. Relations between the OS of the patients and the gene subtypes were revealed by Kaplan–Meier curves. The expression patterns of prognostic DEGs among patients with different gene subtypes or different clinicopathological characteristics were visualized using a heatmap. The differential expression of PRGs between gene subtypes was also evaluated. We then randomly partitioned the TNBC samples from the development cohort into a training set (n=218) and a validation set (n=217) to build a risk model for OS of patients, based on the expression of prognostic DEGs related to pyroptosis. To improve the accuracy of the prediction and resolve the problem of overfitting, LASSO regression was used using the “glmnet” package in R. A 10-fold cross-validation for parameter selection of the LASSO model was performed with the minimum criteria (the value of lambda that gives a minimum mean cross-validated error). The candidate genes were finally selected by a multivariate Cox model regression analysis to generate a risk score in the training set. The OS-related risk score was calculated as:


$$Risk\;Score=\sum_{i=1}^{n}\beta_{i}*Exp_{i}$$

β_i_ and Exp_i_ represented the coefficients and expression levels of each candidate gene. Using the median score as a cutoff value, patients were divided into high- and low-risk subgroups. Kaplan–Meier curves were plotted to analyze survival differences between two groups. Receiver operating characteristic (ROC) curves were generated to assess the model efficacy for 2-, 3-, 5-, 7-, or 10-year OS. Both Kaplan–Meier curves and ROC curves were reanalyzed in the validation set and in the whole development set. Comparisons of risk scores between pyroptosis subtypes and gene subtypes are shown in boxplots. Differentially expressed PRGs were analyzed between high- and low-risk groups. Furthermore, 107 TNBC cases from the GSE58812 dataset were used as an independent external validation cohort to test the risk score model.

### Tumor immunity analysis based on risk score stratification

CIBERSORT was used with the LM22 signature to assess the abundance of 22 immune cell types in the TME of TNBC between the high- and low-risk groups according to the gene expression data (https://cibersort.stanford.edu/). Correlations between the risk score and the fractions of 22 immune cells infiltrating the tumor were analyzed separately. The “ESTIMATE” package was used to calculate the scores for tumor purity, the level of stromal cells, and the level of immune cells present in tumor tissues (https://bioinformatics.mdanderson.org/estimate/). The expression of 58 immune checkpoints was evaluated between two risk groups in all sample sets.

### Somatic mutation and drug sensitivity analysis between risk groups

The somatic mutation information of the high- and low-risk groups was analyzed using the “maftools” package based on the TNBC cases from TCGA-BRCA dataset. TMB and MSI were compared. The MSI was calculated using the scoring system described in the study from Kautto et al., named Microsatellite Analysis for Normal Tumor InStability (MANTIS), which displayed superior performance compared to the previously published computational tools for MSI detection (26). Using the RNA-based stemness score (RNAss) signature from the UCSC XENA browser, we also investigated the relation between cancer stem cells and the risk score (https://xena.ucsc.edu/). To predict the chemotherapeutic response in TNBC patients of different risk groups, we compared the half maximum inhibitory concentration (IC50) of commonly used chemotherapy drugs through the “pRRophetic” package in R.

### Statistical analysis

A two-sided probability value of p<0.05 was considered statistically significant. Data processing and data visualization were performed in RStudio (version 2021.09.1 + 372, https://www.rstudio.com/).

## Results

### Somatic mutation analysis of PRGs

**Figure 1:**
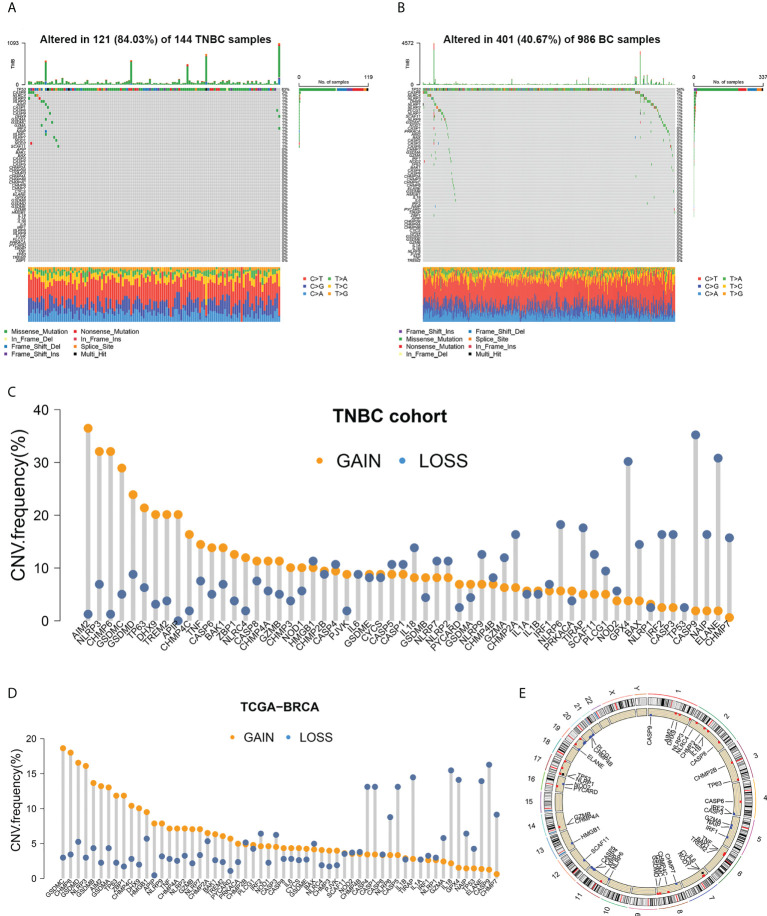
Somatic mutation analysis of PRGs. **(A, B)** Mutation frequencies of 58 PRGs in TNBC samples and all BC samples. **(C, D)** Copy number variations of 58 PRGs in TNBC samples and all BC samples. **(E)** Copy number alteration loci of PRGs on 23 chromosomes in TNBC patients.

The somatic mutation frequencies of 58 investigated PRGs were significantly higher in patients with TNBC (84.03%) than in all BC cohorts (40.67%) (**Figures 1A, B**). TP53 was the most frequently mutated PRG, which was seen in 82.64% of TNBC patients (119/144 samples) and in 34.18% of all BC patients (337/986 samples). CASP8 with somatic mutation was found in 2% of TNBC samples.

Copy number variations (CNVs) were detected in all 58 PRGs in both the TNBC set (**Figure 1C**) and the entire BC set (**Figure 1D**). Among patients with TNBC, AIM2 showed the highest frequency of increase in copy number (with 36.48% of the TNBC samples showing a higher copy number), followed by NLRP3 (32.08%), CHMP6 (32.08%), GSDMC (28.93%), and GSDMD (23.90%). Copy number loss was discovered more frequently in CASP9 (35.22%), ELANE (30.82%), GPX4 (30.19%), NLRP6 (18.24%), and TIRAP (17.61%). Different patterns of CNV were observed in all BC patients, among which GSDMC, CHMP6, GSDMD, NLRP3, and GSDMB exhibited the highest incidence of increased CNV, while CASP9, IL18, TIRAP, GPX4, and ELANE represented PRGs with the most frequent decrease in CNV. Copy number alteration loci of PRGs on chromosomes in TNBC patients are plotted in **Figure 1E**.

### Identification of differentially expressed PRGs and OS-related PRGs

**Figure 2:**
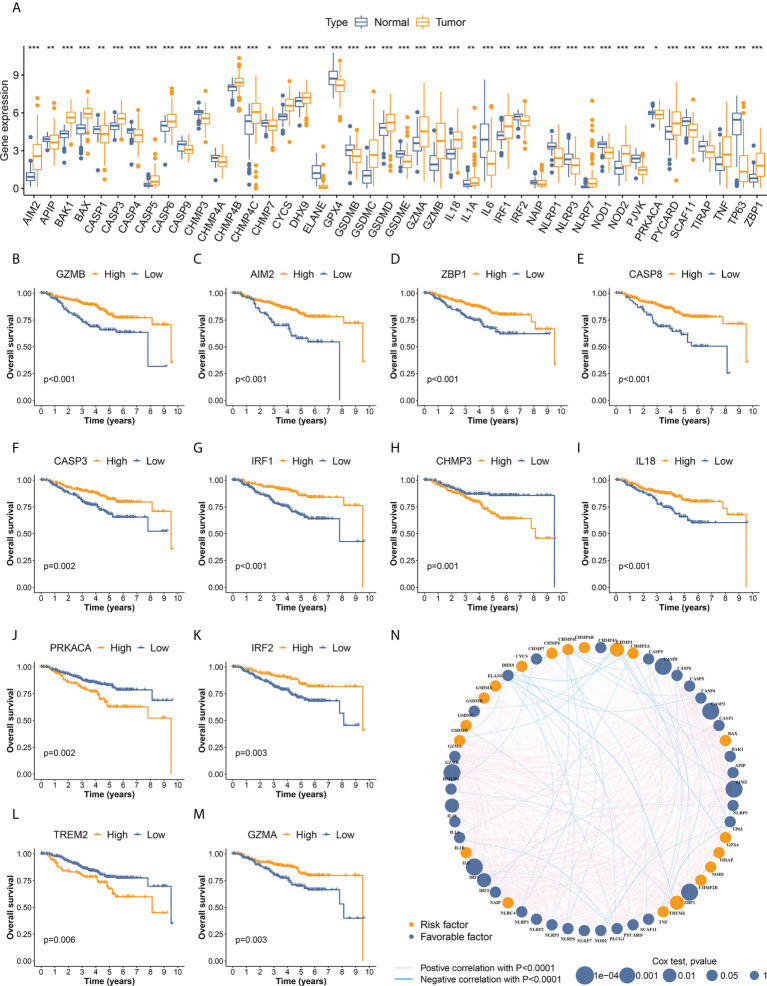
Identification of differentially expressed PRGs and OS-related PRGs in TNBC. **(A)** Differential expression was detected in 44 PRGs between cancer and normal samples. **(B–M)** PRGs significantly associated with overall survival of TNBC patients. Twelve OS-related PRGs showed statistical significance in both survival curves and Cox regression analyses. The other 19 OS-related PRGs with p<0.05 in Kaplan–Meier curves are illustrated in **Supplementary Figure S1**. **(N)** Interactive network and prognostic value of PRGs in TNBC. The thickness of green and pink lines represent the strength of interactive correlation between different PRGs. * p < 0.05, ** p < 0.01, *** p < 0.001.

RNA sequencing data of 162 TNBC samples and 113 normal tissues from TCGA-BRCA were used to investigate differentially expressed PRGs. Different expression levels between cancer and normal samples were detected in 44 of the 58 PRGs (**Figure 2A**). PRGs showing significant CNV gain, including AIM2, GSDMC, and GSDMD, were significantly upregulated in TNBC samples. Similarly, PRGs with high CNV loss frequency, such as CASP9, ELANE, GPX4, and TIRAP, were also markedly downregulated in TNBC. However, there were other PRGs that showed a discrepancy between copy number alterations and mRNA expression (such as NLRP3 and TP63), indicating that CNV could contribute to the regulation of PRG mRNA levels in TNBC, but was not the only factor involved.

A total of 435 individuals with TNBC from the development cohort (162 cases of TCGA-BRCA and 273 of GSE96058) were included in the subsequent survival analysis. The differential expression of 31 PRGs was significantly associated with OS in these patients (**Figures 2B–M**, **Supplementary Figure S2**), suggesting the vital role of pyroptosis in the development of TNBC. The interactive network and the prognostic value of PRGs in TNBC are visualized in **Figure 2N**.

### Classification of pyroptosis subtypes in TNBC

**Figure 3:**
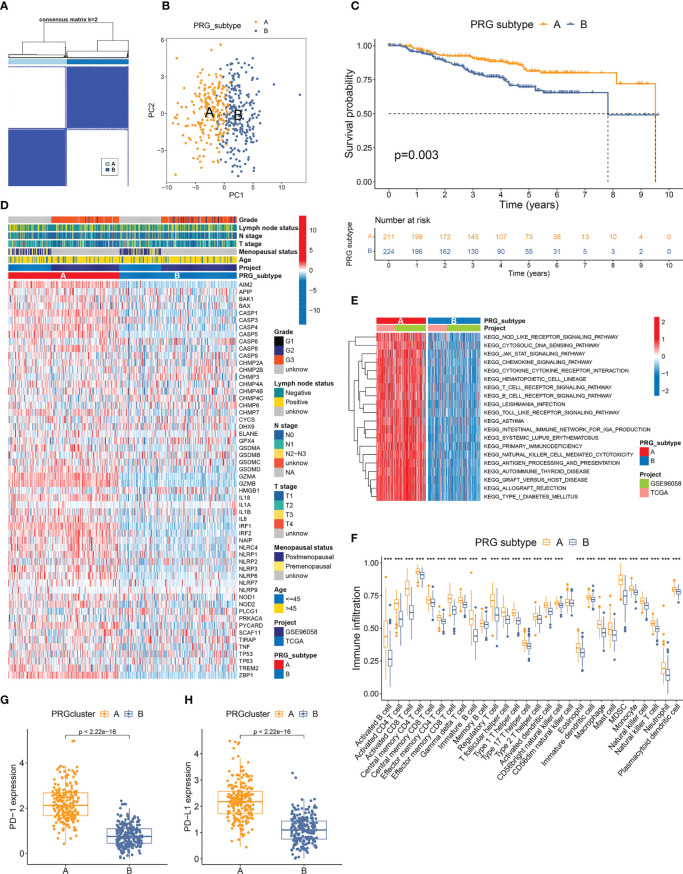
Classification and TME features of pyroptosis subtypes in TNBC. **(A)** Two pyroptosis-related subtypes and their correlation area defined by consensus matrix heatmap. Fifty-six PRGs listed in **Supplementary Table S2** are included in the subtype clustering analyses. **(B)** PCA analysis verified the remarkable differences in pyroptosis transcription profiles between two subtypes. **(C)** TNBC patients defined as PRG subtype A exhibited significantly better OS compared to those from PRG subtype **(B, D)** Comparisons of PRG expression and clinicopathological features between two PRG subtypes. **(E)** GSVA canonical pathway analysis discovered PRG subtype A samples with dramatically higher enrichment in multiple immune-related pathways. **(F)** Twenty-seven immune cell types showed distinctly higher infiltration levels in the microenvironment of subtype **(A, G, H)** Expressions of PD-1 and PD-L1 were remarkably elevated in subtype A. ** p < 0.01, *** p < 0.001.

The PRG expression profile of 435 TNBC samples from the development cohort was recovered for consensus clustering analysis. Fifty-six PRGs, detected in both TCGA-BRCA and GSE96058 datasets, were included in the subtype clustering (**Supplementary Table S2**). The heatmap of the consensus matrix and the CDF curves identified k=2 as the optimal group number, which classified the TNBC samples into group A (n=211) and group B (n=224) (**Figure 3A**, **Supplementary Figure S3**). PCA verified the distinct features of the pyroptosis transcriptomes between two groups (**Figure 3B**). Furthermore, Kaplan–Meier curves revealed markedly better OS in patients of subtype A than those of subtype B (p=0.003) (**Figure 3C**). The heatmap in **Figure 3D** also displayed disparate PRG expression patterns between two pyroptosis subtypes. The clinicopathological characteristics of different subtypes were further compared. The comparative proportions of T1, T2, and T3–T4 tumors between subtypes A and B were 50.98% vs. 39.64%, 41.67% vs. 50.45%, and 7.35% vs. 9.91%, respectively (p=0.040), suggesting that tumors of subtype A tended to have a lower T stage (**Figure 3D**).

### TME characterization of pyroptosis subtypes

GSVA analysis of the canonical pathway of 435 TNBC samples distinguished subtype A as the subtype with dramatically higher enrichment in activated immune pathways, including NOD-like receptor signaling, cytosolic DNA sensing pathway, JAK-STAT signaling pathway, chemokine signaling pathway, cytokine–cytokine receptor interaction, antigen processing and presentation, T-cell receptor signaling, and B-cell receptor signaling pathway (**Figure 3E**). ssGSEA showed that 27 of the total 28 immune cell types investigated had markedly higher infiltration levels in the TME of subtype A compared to that of subtype B (**Figure 3F**). Furthermore, the expression of both PD-1 and PD-L1 was also remarkably elevated in subtype A samples compared to those of subtype B (**Figures 3G, H**). These results suggested a stronger immunogenicity and antitumor response in patients with TNBC of subtype A.

### Identification of DEGs among pyroptosis subtypes and development of DEG-based gene subtypes

**Figure 4:**
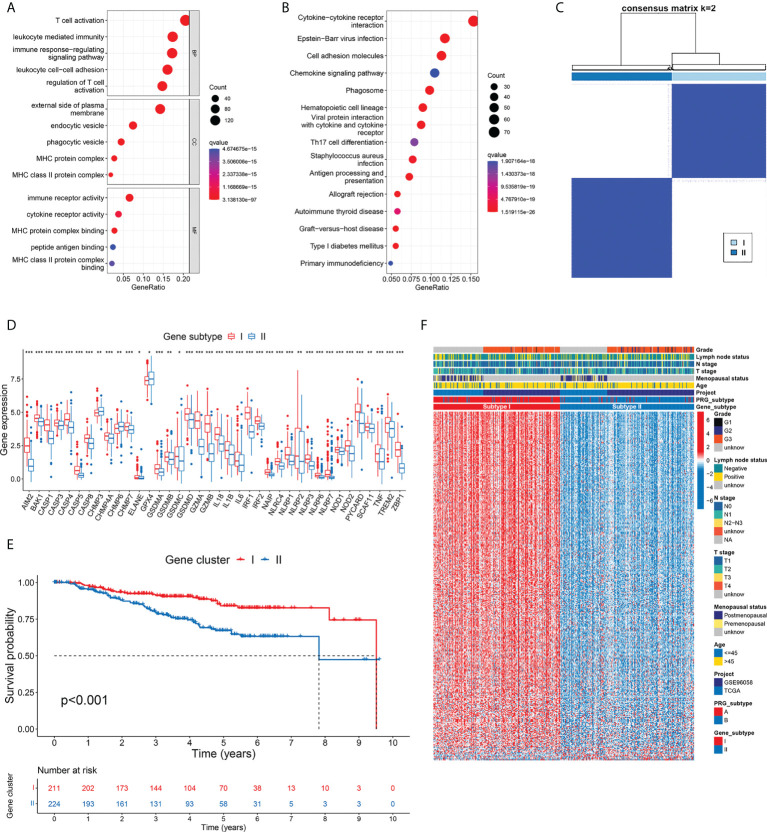
Development of gene subtypes based on differentially expressed genes between PRG subtypes. **(A**, **B)** GO and KEGG enrichment of DEGs identified between two PRG subtypes. **(C)** Consensus matrix heatmap defined two gene subtypes based on 346 prognostic DEGs. **(D)** Thirty-eight PRGs were differentially expressed between two gene subtypes. **(E)** TNBC patients defined as gene subtype I showed significantly better OS. **(F)** Comparison of gene expression and phenotype features between two gene subtypes. * p < 0.05, ** p < 0.01, *** p < 0.001.

To disclose the biological differences related to different subtypes of pyroptosis, we identified 844 DEGs between subtype A and B tumors (**Supplementary Table S3**). The GO annotations of DEGs in terms of their biological process (BP), cellular component (CC), and molecular function (MF) are summarized in **Figure 4A**. The top-ranked GO terms were mostly related to hemopoietic cells and immunological processes, such as T-cell activation, leukocyte-mediated immunity, external side of the plasma membrane, immune receptor activity, and cytokine receptor activity. The pathway in which most DEGs were involved was the cytokine–cytokine receptor interaction, followed by multiple immune-related pathways including cell adhesion molecules and the chemokine signaling pathway (**Figure 4B**). The results of functional enrichment highlighted the potential role of pyroptosis in the immune response of TNBC.

To further differentiate TNBC patients with varied DEG patterns, we screened 346 genes that were significantly associated with OS from the 844 DEGs related to pyroptosis by univariate Cox regression (**Supplementary Table S4**). Among them, 337 genes were favorable predictors, while 9 genes were risk factors. According to the 346 prognostic DEGs, we classified patients into two gene subtypes using the consensus clustering algorithm (**Figure 4C**; **Supplementary Figure S4**). Gene subtype I included 211 patients, while the other 224 cases were defined as gene subtype II. Consistent with the pyroptosis-based classification, differential expression of PRG was observed between gene subtypes (**Figure 4D**). Furthermore, survival analysis showed a worse OS in patients with gene subtype II tumors compared to those with gene cluster I tumors (p<0.001) (**Figure 4E**). The comparison of gene expression and phenotype characteristics between two gene subtypes is illustrated in **Figure 4F**. The pyroptosis subtypes and the gene subtypes exhibited high concordance in the patient distribution. In gene subtype I, 90.05% of the samples were from pyroptosis subtype A, while 90.63% of the samples from gene subtype II were from pyroptosis subtype B. As shown in the heatmap, a large proportion of DEGs, most of which were favorable predictors of OS, showed particularly higher expression in gene subtype I (consistent with the better survival outcome in subtype I patients). Similarly, subtype I samples had lower T stage (p=0.028).

### Construction of a risk score based on pyroptosis-related prognostic DEGs

**Figure 5:**
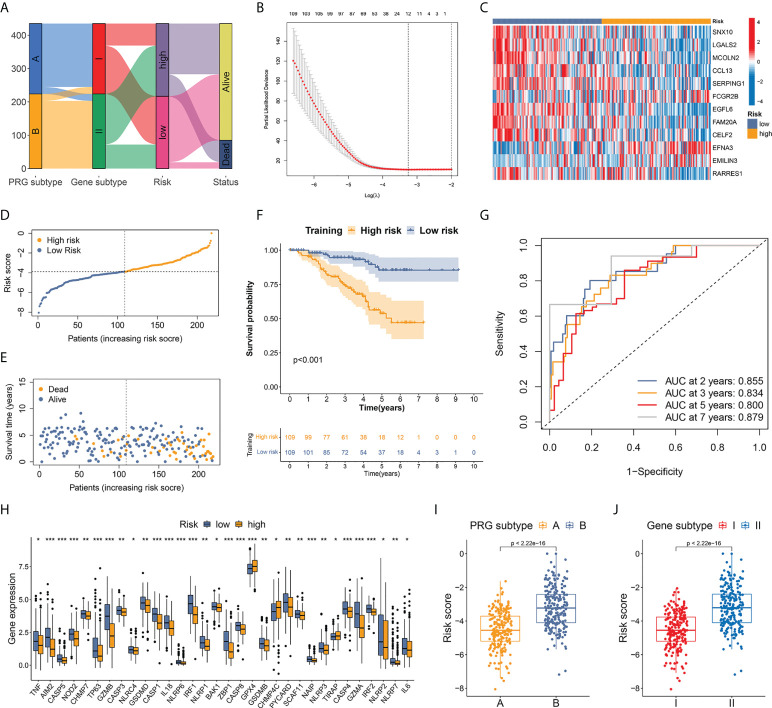
Establishment of a risk score based on pyroptosis-related prognostic DEGs. **(A)** Alluvial diagram of patients’ distribution between different subtypes, risk groups, and vital status. **(B)** OS-related DEGs identified by univariate Cox analysis were screened by LASSO regression. **(C)** Expression heatmap of the 12 hub DEGs between two risk groups. **(D)** Risk score distribution in patients from training set. A median score of −3.898 was defined as the cutoff value. **(E)** Vital status plot showed higher death rate in patients from high-risk group in training set. **(F)** TNBC patients from high-risk group had markedly worse OS than low-risk patients. **(G)** AUC of ROC curves showed good performance of the 12-gene-based risk score in predicting 2-, 3-, 5-, and 7-year OS for patients in training set. **(H)** Differential expression of 32 PRGs between high- and low-risk groups. **(I, J)** Patients from both pyroptosis subtype A and gene subtype I, which represented activated anti-tumor immunity and better OS, had significantly lower risk score compared to subtype B and subtype II. * p < 0.05, ** p < 0.01, *** p < 0.001.

Four hundred thirty-five cases from the development cohort (composed of TCGA-BRCA and GSE96058) were included in the risk model exploration. The alluvial diagram in **Figure 5A** shows the patients’ distribution between different subtypes, risk groups, and vital status. The patients were randomly partitioned into a training set (n=218) and an internal validation set (n=217). Based on the survival outcomes of the patients and the expression of 346 prognostic DEGs related to pyroptosis, we selected 12 candidate genes by LASSO regression and cross-validation (**Figure 5B**; **Supplementary Figure S5**). Using multivariate Cox regression analysis, we finally used the 12 DEGs (CCL13, CELF2, EFNA3, EGFL6, EMILIN3, FAM20A, FCGR2B, LGALS2, MCOLN2, RARRES1, SERPING1, and SNX10) and established the risk score with their coefficients and expression levels. Nine DEGs (CCL13, CELF2, EGFL6, FAM20A, LGALS2, MCOLN2, RARRES1, SERPING1, and SNX10) were favorable predictors, while the other three were high-risk factors (EFNA3, EMILIN3, and FCGR2B) (**Supplementary Table S5**). The expression heatmap of the 12 DEGs between the two risk groups is illustrated in **Figure 5C**.

A median score of −3.898 in the training set was defined as the cutoff value to distinguish high- and low-risk patients (**Figure 5D**). The vital status plot revealed that patients in the high-risk group had a higher death rate than those in the low-risk group (**Figure 5E**). The Kaplan–Meier curves confirmed significantly worse OS in high-risk individuals (**Figure 5F**): comparison of the 2-year OS rates between the high- and low-risk groups was 82.71% vs. 96.92%, while the 5-year OS rates were 53.72% vs. 85.45% (p<0.001). In ROC curve analysis, the areas under the curve (AUCs) for the prediction of 2-, 3-, 5-, and 7-year OS were 0.855, 0.834, 0.800, and 0.879, respectively (**Figure 5G**).

Risk stratification was repeated in the internal validation set using the median score of the training set (**Supplementary Figure S6**). Vital status and Kaplan–Meier curves verified favorable survival outcomes in the low-risk group with p-value <0.001 (**Supplementary Figures S7**, **8**). The AUCs for the 2-, 3-, 5-, and 10-year OS prediction were 0.644, 0.719, 0.736, and 0.757, respectively, in the internal validation set (**Supplementary Figure S9**). Furthermore, differential expression of 32 PRGs was detected between high- and low-risk groups among all patients in the development cohort (**Figure 5H**). Among the 435 individuals in the entire development cohort, patients from both pyroptosis subtype A and gene subtype I, which represented activated cancer immunity and better OS, had a markedly lower risk score compared to subtype B and subtype II, respectively (p<0.001 in both tests) (**Figures 5I, J**), suggesting that the lower risk score could be associated with upregulated immune defense in the microenvironment of TNBC.

To further validate the prognostic value of the risk model, we performed the risk score calculation in an independent external validation cohort (GSE58812). A better long-term OS was observed in low-risk cases (p=0.025, **Supplementary Figure S10**), who had a significantly higher 5- and 10-year OS rate compared to high-risk patients (81.26% vs. 69.47 at 5 years, 78.80% vs. 59.47% at 10 years). The AUC for the prediction of OS at 3, 5, 7, and 10 years were 0.714, 0.766, 0.721, and 0.725, respectively (**Supplementary Figure S11**), demonstrating good performance of the 12-DEG-based risk score in the prediction of long-term prognosis for patients with TNBC.

### Evaluation of tumor immune microenvironment based on risk stratification

**Figure 6:**
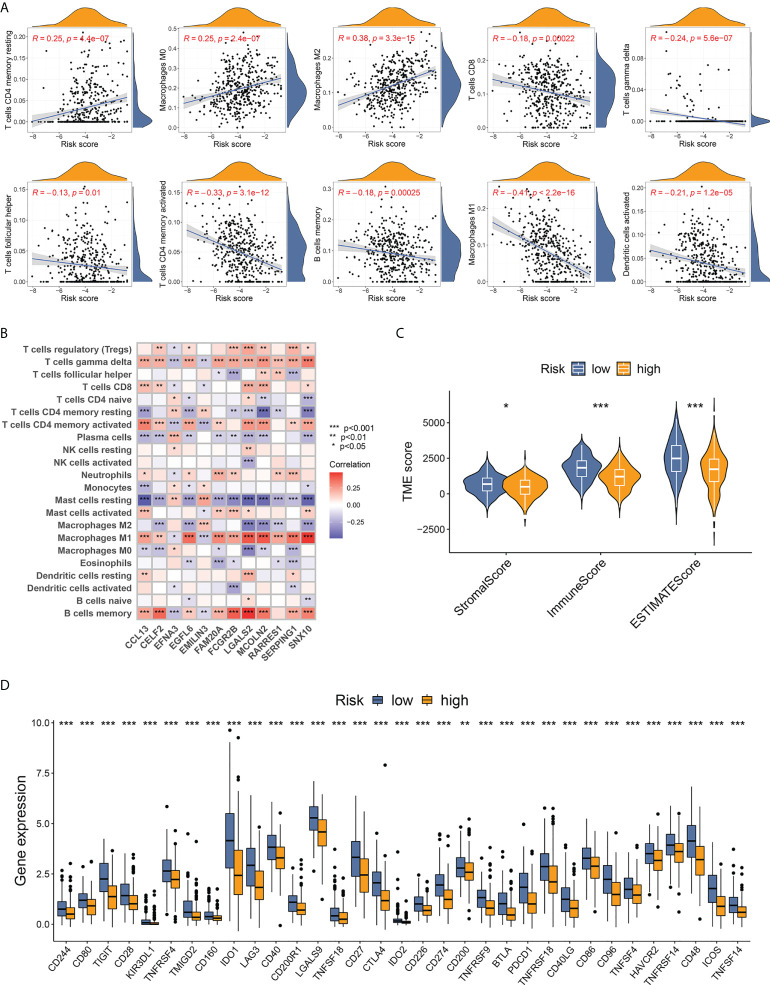
Evaluation of TME features based on the 12-DEG risk score. **(A)** Correlation between immune cell infiltrating fractions and the 12-DEG-based risk scores in TNBC samples. **(B)** Correlation between the 12 DEGs from the scoring system and the abundance of 22 analyzed immune cell types. **(C)** TNBC samples from low-risk patients showed higher stromal, immune, and ESTIMATE scores, indicating lower tumor purity with more stromal cells and immune cells in TME. **(D)** A total of 33 immune checkpoints were upregulated in low-risk TNBC patients. * p < 0.05, ** p < 0.01, *** p<0.001.

To explore tumor immunity and the microenvironment in TNBC from different risk groups, we analyzed the correlation between risk score and immune cell abundance using the CIBERSORT algorithm. The risk score was positively related to the fraction of three non-activated or pro-tumorigenic cell types (resting CD4+ memory T cells, M0 macrophages, and M2 macrophages) and was negatively correlated with seven types of antitumor immune cell types (CD8+ T cells, gamma delta T cells, follicular helper T cells, activated CD4+ memory T cells, memory B cells, M1 macrophages, and activated dendritic cells) (**Figure 6A**). The 22 immune cell types analyzed exhibited a statistically significant correlation with at least one of the 12 DEGs of the scoring model (**Figure 6B**). The results of low-risk score samples with high abundance of antitumor immune cells were consistent with the finding that low-risk patients were more likely to exist in immune-activated subtypes.

The “ESTIMATE” package was utilized to calculate the TME score, containing a stromal score (capturing the presence of stromal cells in tumor tissue), an immune score (representing the infiltration of immune cells), and an ESTIMATE score (a combination of stromal and immune scores that inferred tumor purity). TNBC patients from the low-risk group scored higher in all three fields (**Figure 6C**), indicating a lower tumor purity with higher infiltration of both stromal cells and immune cells. Furthermore, the expression of immune checkpoints between different risk groups was compared. Thirty-three immune checkpoints, including PD-1 (PDCD1), PD-L1 (CD274), CTLA-4, and LAG3, showed markedly upregulated levels in low-risk samples (**Figure 6D**), implying better response to immunotherapy for low-risk patients with TNBC.

### MSI and mutation analysis

**Figure 7:**
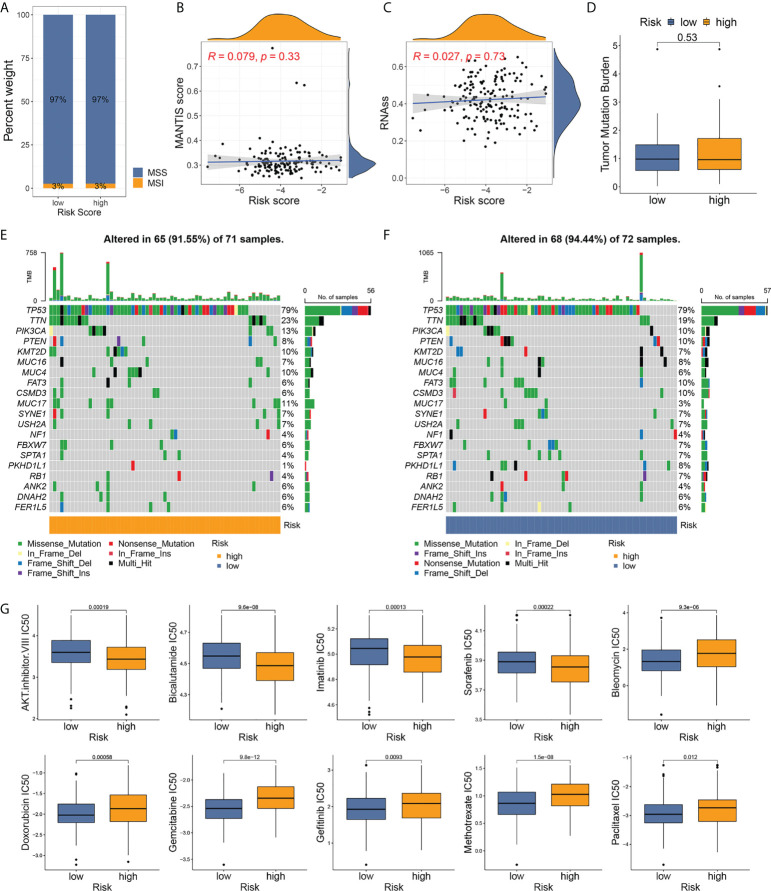
Analyses of microsatellite instability, somatic mutation, and drug susceptibility based on risk stratification. **(A)** Distribution of MSI incidence among high- and low-risk patients. **(B)** Correlation between MSI score and risk score of TNBC samples. **(C)** Correlation between RNA stemness score and risk score. **(D)** Comparison of TMB between risk groups. **(E, F)** Somatic mutation frequencies in high- and low-risk TNBC tumors. **(G)** Drug susceptibility analyses (IC50) of commonly used chemotherapy reagents between high- and low-risk patients.

The MSI status was investigated between the risk groups. More than 95% of patients with TNBC were defined as microsatellite stable (MSS) regardless of risk stratification (**Figure 7A**), consistent with previous reports that MSI incidence was rarely observed in BC compared to other types of cancer (12, 27). The MSI score values did not have statistical correlation with the pyroptosis-related risk scores (**Figure 7B**, p=0.33), and no difference in the distribution of the risk score was observed between the MSI and MSS cases (**Supplementary Figure S12**). A trend of higher RNAss was observed with increasing risk score, while no statistical significance was observed (**Figure 7C**).

The TMB level was low in patients with TNBC and in all patients with BC (**Supplementary Figure S13**). No differences in TMB were observed between the two groups (**Figure 7D**). The waterfall graphs (**Figures 7E, F**) represented the characteristics of the somatic mutation between high- and low-risk individuals from the development cohort. A total of 143 patients with mutational data from TCGA-BRCA program were involved in the analysis. TP53, TTN, PIK3CA, PTEN, KMT2D, MUC16, MUC4, FAT3, CSMD3, and MUC17 ranked the top 10 most frequently mutated genes in both groups. Among them, a higher mutation rate of TTN, PIK3CA, KMT2D, MUC4, and MUC17 was observed in high-risk patients.

### Drug sensitivity analysis

To select the optimal treatment for patients with TNBC, we evaluated the susceptibility of commonly used chemotherapy drugs among high- and low-risk cases. Patients at high risk were more sensitive to the AKT inhibitor VIII, bicalutamide, imatinib, and sorafenib, while low-risk patients in low-risk groups had lower IC50 for bleomycin, doxorubicin, gemcitabine, gefitinib, methotrexate, and paclitaxel (**Figure 7G**). The drug susceptibility results implied that the classic regimen of chemotherapy based on taxane or anthracycline for BC could be avoided in patients with high-risk TNBC.

## Discussion

Recent studies have unraveled the crucial role of genes associated with pyroptosis in breast tumor immunity, most of which elucidate the pro- or antitumor mechanism of a single PRG (28–30). Few studies have focused on the collaborative effects of multiple PRGs on the immune microenvironment specifically in TNBC. In the present study, we disclosed genetic alterations and expression patterns of more than 50 PRGs in patients with TNBC. It is worth mentioning that TP53 gene, mutated in over half of all human malignancies (31), showed frequent mutation (82.64%) in TNBC in this study, which was concordant with previous research results that TNBC had the highest prevalence of TP53 mutation among all breast cancers (32). However, the somatic mutation of TP53 did not cause its alteration in measured expression levels in TNBC compared to normal tissues (as shown in **Figure 2A**, TP53 was not among the differentially expressed PRGs). The role of TP53 in pyroptosis has not been fully studied. Expression of GSDME could be induced by p53, a tumor suppressor protein encoded by TP53 (33). Zhang et al. also discovered that the upregulation of p53 in non-small-cell lung cancer could prompt pyroptosis and produce antitumor effects (34). Further studies are needed to explore the association between TP53 mutation and pyroptosis in TNBC.

Based on PRG expression profiling, we identified two pyroptosis subtypes that harbored distinct characteristics of the TME and survival outcomes. Patients with pyroptosis subtype A had a lower T stage and notably better OS compared to patients with subtype B tumors. Additionally, subtype A TNBC samples exhibited fully activated immune microenvironments, with significantly higher infiltration levels of 27 types of immune cells, including B cells, CD4+ and CD8+ T cells, gamma delta T cells, and macrophages. Activation of multiple immune-related pathways was also detected in subtype A tumors, including NOD-like receptor signaling, chemokine signaling pathway, cytokine–cytokine receptor interaction, antigen processing and presentation, T-cell receptor signaling, and B-cell receptor signaling pathway. Furthermore, transcriptome profiling differed dramatically between pyroptosis subtypes, and DEGs identified between two subtypes were involved in immunological processes such as T-cell activation and immune receptor activity. Using the expression of DEGs associated with survival, we further classified TNBC patients into two gene subtypes. More than 97% of OS-related DEGs were survival benefiting, and most were distinctively elevated in gene subtype I. Furthermore, gene subtype I showed a high concordance in patient distribution with pyroptosis subtype A and a longer OS compared to subtype II. The above findings revealed that PRG alterations were closely related to the activation of the cancer immune microenvironment and the intensity of the antitumor response, which could lead to significant changes in OS of TNBC patients.

We utilized the pyroptosis-related prognostic DEGs and constructed a 12-gene-based score with reliable performance and robust efficacy in risk prediction. Remarkable differences were observed between high- and low-risk patients with respect to short- and long-term prognosis, TME characteristics, immune checkpoint expression, somatic mutation patterns, MSI scores, and susceptibility to chemotherapy.

Innate and adaptive immune cells in the TME modulate cancer progression and the therapeutic response (35). Evidence has shown the vital role of pyroptosis in maintaining the diversity and complexity of TME in breast tumors (36). Meanwhile, cytotoxic T cells and helper T cells are required for pyroptosis-induced tumor regression (16). In this study, TNBC samples from different pyroptosis subtypes and risk groups differed distinctly in the degree of immune cell infiltration. The presence of tumor-infiltrating lymphocytes (TILs) is predictive of a better response to immunotherapy and a favorable prognosis in BC (37–40). CD8+ T-cell infiltration in BC is independently associated with a reduced relative risk of cancer-related death (41), while TNBCs with CD8 positivity have greater possibilities to benefit from immunotherapy (42). These findings correspond to our results of more CD8+ T cells in the TME of pyroptosis subtype A and low-risk group that had higher expression of PD-1 and PD-L1 and better OS. Functional follicular helper T cells (Tfh) oriented by T-helper 1 (Th1) cells can promote humoral and cytotoxic immune responses in human breast cancer (43), and the presence of CXCL13-producing Tfh cells in tertiary lymphoid structures (TLS) of breast tumors robustly predicts positive clinical outcomes (44). Gamma delta T cells target tumor cells in TNBC (45), and their numbers in TME are positively related to the immunotherapy response of patients with advanced BC (46). Activated CD4+ memory T cells are also related to better survival in different cancer types (47–49). In this study, the above three types of T cells showed higher infiltration in subtype A patients and patients with low scores, indicating the potential of our pyroptosis-related risk score to identify TNBCs with greater sensitivity to immunotherapy. Tumor-infiltrating B cells (TIL-B) are another key component of TILs. TIL-B functions in anti-BC responses through antibody production, Th1 responses, and antigen presentation (50). CXCL13-producing Tfh cells can promote memory B-cell differentiation, thus facilitating humoral immune defense in BC (51). Hu et al. reported that memory B-cell transcription signatures were associated with improved overall and disease-free survival in patients with BC (52). Furthermore, memory B cells within TLS are associated with an improved response to immune checkpoint blockade (53). The sustained presence of memory B cells in TNBC is required for the control of myeloid-derived suppressor cells and for the durable efficacy of ICI treatment (54). Consistent with existing evidence, we observed a higher proportion of memory B cells within subtype A tumors and low-score samples, suggesting the role of memory B cells as an indicator of prognosis and ICI response in TNBC. Subpopulations of macrophage exhibit different functions in TME. Macrophages of the M1 phenotype, reprogrammed with the D2 dopamine receptor, can induce pyroptosis of GSDME-executed breast tumor cells (55). In our study, patients in the low-risk group, who had increased expression of nearly 30 PRGs compared to high-risk patients, showed a higher abundance of M1 macrophages in TME, suggesting the crosstalk between pyroptosis and macrophage-mediated cancer immunity. In contrast, M2 macrophages, the other polarized phenotype, are immunosuppressive and pro-tumorigenic. Zhang et al. revealed that TNBC cells induced an elevation of YAP expression in macrophages, which polarized macrophages to the M2 phenotype and increased the pro-metastatic potential of cancer cells *via* MCP-1/CCR2 pathway (56). In line with previous studies, we discovered a positive correlation between the increase in the risk score and the proportion of M2 macrophages in the immune microenvironment of TNBC, while the presence of M1 macrophages was negatively associated with the value of the risk score in patients. Dendritic cells, which are critical for T-cell activation and immunosurveillance in BC (57), also showed a lower infiltration rate as the risk score increased according to our findings. The correlation between immune cell abundance and pyroptosis-related risk stratification in our study implied the pivotal role of pyroptosis in shaping an activated antitumor microenvironment in TNBC.

BC has been defined as immunologically “cold” due to low T-cell infiltration and inefficient T-cell priming compared to other malignancies (58, 59). Non-synonymous DNA mutations, capable of increasing the presence of neoantigens and inducing the immune cell response, are also found with relatively low burden in breast tumors (60, 61). Despite the non-immunogenic nature, the magnitude of TILs varies within and between BC subtypes (37, 62). Increasing evidence has revealed that TNBC patients, previously termed the most aggressive subtype with a poor prognosis, have a higher abundance of TIL than non-TNBC patients, which could contribute to better survival after ICI treatment (37, 63, 64). However, extensive heterogeneity of the TME and biological behavior still exists within the TNBC cell population. In the present study, we identified individuals with “hot” immune status from TNBC patients based on the clustering of pyroptosis-related subtypes and stratification of risk scores. In addition to increased infiltration of tumor suppressor immune cells in TME, lower tumor purity calculated by the ESTIMATE algorithm was also observed in low-risk patients. A favorable OS has been found in patients with a high stromal score and an immune score in other cancer types (65). Similarly, our findings showed that TNBCs with low-risk score presented a higher immune score and lower tumor purity than high-risk cases. Hou et al. has discovered that PD-L1 can mediate GSDMC expression and trigger pyroptosis in BC cells (66). Beyond that, little is known about the interaction between pyroptosis and PD-1/PD-L1 function. In this study, we demonstrated higher expression of PD-1 and PD-L1 in both the pyroptosis subtype A and low-risk group. In addition, there were an additional 31 immune checkpoints showing a significantly higher level in low-risk-score TNBC, suggesting the ability of the pyroptosis-related risk score to predict the response of ICI therapy in TNBC. The results also provided a potential strategy of blocking other immune checkpoints for early-stage TNBC, including anti-CTLA-4 reagents, which are currently being used in mouse experiments and clinical trials for advanced BC (10, 67, 68). TMB and MSI predict a stronger response to ICI and prolonged survival in colorectal cancer, non-small cell lung cancer, and melanoma (69–72). However, no differences in TMB or MSI were detected between two risk groups, possibly due to the rare incidence of non-synonymous mutations and mismatch repair in BC patients (11, 12, 27). In line with existing research, the low-TMB/low-MSI nature of BC discovered in our study suggested that better predictive biomarkers for response to immunotherapy are needed for TNBC patients. Our findings also indicated the capacity of our scoring model to identify immune “hot” cases among the low-TMB/low-MSI TNBC population.

The major limitation of this study was that the data used for the analyses were derived from public databases, and some clinicopathological information, including patients’ history of systemic treatment, was unavailable. Prospective studies and exploratory experiments are needed to further validate the efficacy of this pyroptosis-based risk stratification model.

## Data availability statement

The datasets presented in this study can be found in online repositories. The names of the repository/repositories and accession number(s) can be found in the article/**Supplementary Material**.

## Author contributions

LL and WZ designed and organized the study. LL, QW, and CX carried out bioinformatics analyses and drew figures. LL drafted the manuscript. QW and MD participated in the manuscript editing. All authors contributed to the article and approved the submitted version.

## Funding

This work was supported by Zhejiang Provincial Natural Science Foundation of China (No. LQ19H160034) and Medical Health Science and Technology Project of Zhejiang Provincial Health Commission, China (No. 2018KY485).

## Conflict of interest

The authors declare that the research was conducted in the absence of any commercial or financial relationships that could be construed as a potential conflict of interest.

## Publisher’s note

All claims expressed in this article are solely those of the authors and do not necessarily represent those of their affiliated organizations, or those of the publisher, the editors and the reviewers. Any product that may be evaluated in this article, or claim that may be made by its manufacturer, is not guaranteed or endorsed by the publisher.

## References

[1] Bianchini G De Angelis C Licata L Gianni L. Treatment landscape of triple-negative breast cancer - expanded options, evolving needs. Nat Rev Clin Oncol (2022) 19(2):91–113. doi: 10.1038/s41571-021-00565-2 34754128

[2] Barroso-Sousa R Jain E Cohen O Kim D Buendia-Buendia J Winer E. Prevalence and mutational determinants of high tumor mutation burden in breast cancer. Ann Oncol (2020) 31(3):387–94. doi: 10.1016/j.annonc.2019.11.010 32067680

[3] Cortes J Cescon DW Rugo HS Nowecki Z Im SA Yusof MM. Pembrolizumab plus chemotherapy versus placebo plus chemotherapy for previously untreated locally recurrent inoperable or metastatic triple-negative breast cancer (Keynote-355): A randomised, placebo-controlled, double-blind, phase 3 clinical trial. Lancet (2020) 396(10265):1817–28. doi: 10.1016/s0140-6736(20)32531-9 33278935

[4] Schmid P Cortes J Pusztai L McArthur H Kummel S Bergh J. Pembrolizumab for early triple-negative breast cancer. N Engl J Med (2020) 382(9):810–21. doi: 10.1056/NEJMoa1910549 32101663

[5] Miles D Gligorov J Andre F Cameron D Schneeweiss A Barrios C. Primary results from Impassion131, a double-blind, placebo-controlled, randomised phase iii trial of first-line paclitaxel with or without atezolizumab for unresectable locally Advanced/Metastatic triple-negative breast cancer. Ann Oncol (2021) 32(8):994–1004. doi: 10.1016/j.annonc.2021.05.801 34219000

[6] Schmid P Adams S Rugo HS Schneeweiss A Barrios CH Iwata H. Atezolizumab and nab-paclitaxel in advanced triple-negative breast cancer. N Engl J Med (2018) 379(22):2108–21. doi: 10.1056/NEJMoa1809615 30345906

[7] Kassardjian A Shintaku PI Moatamed NA. Expression of immune checkpoint regulators, cytotoxic T lymphocyte antigen 4 (Ctla-4) and programmed death-ligand 1 (Pd-L1), in female breast carcinomas. PloS One (2018) 13(4):e0195958. doi: 10.1371/journal.pone.0195958 29672601 PMC5909602

[8] Santa-Maria CA Kato T Park JH Kiyotani K Rademaker A Shah AN. A pilot study of durvalumab and tremelimumab and immunogenomic dynamics in metastatic breast cancer. Oncotarget (2018) 9(27):18985–96. doi: 10.18632/oncotarget.24867 PMC5922371 29721177

[9] McArthur HL Comen EA Bryce Y Solomon SB Leal JHS Abaya CD. A single-arm, phase 2 study of perioperative ipilimumab, nivolumab, and cryoablation in women with hormone receptor-negative, Her2-negative, early-Stage/Resectable breast cancer. J Clin Oncol (2022) 40(16_suppl):TPS617–TPS. doi: 10.1200/JCO.2022.40.16_suppl.TPS617

[10] Kyte JA Andresen NK Russnes HG Fretland SØ Falk RS Lingjærde OC. Icon: A randomized phase iib study evaluating immunogenic chemotherapy combined with ipilimumab and nivolumab in patients with metastatic hormone receptor positive breast cancer. J Transl Med (2020) 18(1):269. doi: 10.1186/s12967-020-02421-w 32620163 PMC7333428

[11] Ding H Zhao J Zhang Y Wang G Cai S Qiu F. Tumor mutational burden and prognosis across pan-cancers. Ann Oncol (2018) 29:viii16–viii7. doi: 10.1093/annonc/mdy269.055

[12] Ding L Bailey MH Porta-Pardo E Thorsson V Colaprico A Bertrand D. Perspective on oncogenic processes at the end of the beginning of cancer genomics. Cell (2018) 173(2):305–20.e10. doi: 10.1016/j.cell.2018.03.033 29625049 PMC5916814

[13] Broz P Pelegrin P Shao F. The gasdermins, a protein family executing cell death and inflammation. Nat Rev Immunol (2020) 20(3):143–57. doi: 10.1038/s41577-019-0228-2 31690840

[14] Liu X Zhang Z Ruan J Pan Y Magupalli VG Wu H. Inflammasome-activated gasdermin d causes pyroptosis by forming membrane pores. Nature (2016) 535(7610):153–8. doi: 10.1038/nature18629 PMC5539988 27383986

[15] Xia X Wang X Cheng Z Qin W Lei L Jiang J. The role of pyroptosis in cancer: Pro-cancer or pro-"Host"? Cell Death Dis (2019) 10(9):650. doi: 10.1038/s41419-019-1883-8 31501419 PMC6733901

[16] Wang Q Wang Y Ding J Wang C Zhou X Gao W. A bioorthogonal system reveals antitumour immune function of pyroptosis. Nature (2020) 579(7799):421–6. doi: 10.1038/s41586-020-2079-1 32188939

[17] Zhang Z Zhang Y Xia S Kong Q Li S Liu X. Gasdermin e suppresses tumour growth by activating anti-tumour immunity. Nature (2020) 579(7799):415–20. doi: 10.1038/s41586-020-2071-9 PMC7123794 32188940

[18] Kim MS Lebron C Nagpal JK Chae YK Chang X Huang Y. Methylation of the Dfna5 increases risk of lymph node metastasis in human breast cancer. Biochem Biophys Res Commun (2008) 370(1):38–43. doi: 10.1016/j.bbrc.2008.03.026 18346456 PMC3094717

[19] Hergueta-Redondo M Sarrio D Molina-Crespo A Megias D Mota A Rojo-Sebastian A. Gasdermin-b promotes invasion and metastasis in breast cancer cells. PLoS One (2014) 9(3):e90099. doi: 10.1371/journal.pone.0090099 24675552 PMC3967990

[20] Molina-Crespo A Cadete A Sarrio D Gamez-Chiachio M Martinez L Chao K. Intracellular delivery of an antibody targeting gasdermin-b reduces Her2 breast cancer aggressiveness. Clin Cancer Res (2019) 25(15):4846–58. doi: 10.1158/1078-0432.CCR-18-2381 31064780

[21] Scarpitta A Hacker UT Buning H Boyer O Adriouch S. Pyroptotic and necroptotic cell death in the tumor microenvironment and their potential to stimulate anti-tumor immune responses. Front Oncol (2021) 11:731598. doi: 10.3389/fonc.2021.731598 34490126 PMC8417056

[22] Daley D Mani VR Mohan N Akkad N Pandian G Savadkar S. Nlrp3 signaling drives macrophage-induced adaptive immune suppression in pancreatic carcinoma. J Exp Med (2017) 214(6):1711–24. doi: 10.1084/jem.20161707 PMC5461004 28442553

[23] Li X-Y Zhang L-Y Li X-Y Yang X-T Su L-X. A pyroptosis-related gene signature for predicting survival in glioblastoma. Front Oncol (2021) 11:697198. doi: 10.3389/fonc.2021.697198 34485134 PMC8416108

[24] Chen W Zhang W Zhou T Cai J Yu Z Wu Z. A newly defined pyroptosis-related gene signature for the prognosis of bladder cancer. Int J Gen Med (2021) 14:8109–20. doi: 10.2147/IJGM.S337735 PMC8594790 34803395

[25] Xu D Ji Z Qiang L. Molecular characteristics, clinical implication, and cancer immunity interactions of pyroptosis-related genes in breast cancer. Front Med (Lausanne) (2021) 8:702638. doi: 10.3389/fmed.2021.702638 34589498 PMC8473741

[26] Kautto EA Bonneville R Miya J Yu L Krook MA Reeser JW. Performance evaluation for rapid detection of pan-cancer microsatellite instability with mantis. Oncotarget (2017) 8(5):7452–63. doi: 10.18632/oncotarget.13918 PMC5352334 27980218

[27] Wen YH Brogi E Zeng Z Akram M Catalano J Paty PB. DNA Mismatch repair deficiency in breast carcinoma: A pilot study of triple-negative and non-Triple-Negative tumors. Am J Surg Pathol (2012) 36(11):1700–8. doi: 10.1097/PAS.0b013e3182627787 22992699

[28] Faria SS Costantini S de Lima VCC de Andrade VP Rialland M Cedric R. Nlrp3 inflammasome-mediated cytokine production and pyroptosis cell death in breast cancer. J BioMed Sci (2021) 28(1):26. doi: 10.1186/s12929-021-00724-8 33840390 PMC8040227

[29] An H Heo JS Kim P Lian Z Lee S Park J. Tetraarsenic hexoxide enhances generation of mitochondrial ros to promote pyroptosis by inducing the activation of caspase-3/Gsdme in triple-negative breast cancer cells. Cell Death Dis (2021) 12(2):159. doi: 10.1038/s41419-021-03454-9 33558527 PMC7870965

[30] Tamura Y Morikawa M Tanabe R Miyazono K Koinuma D. Anti-pyroptotic function of tgf-beta is suppressed by a synthetic dsrna analogue in triple negative breast cancer cells. Mol Oncol (2021) 15(5):1289–307. doi: 10.1002/1878-0261.12890 PMC8096786 33342034

[31] Marei HE Althani A Afifi N Hasan A Caceci T Pozzoli G. P53 signaling in cancer progression and therapy. Cancer Cell Int (2021) 21(1):703. doi: 10.1186/s12935-021-02396-8 34952583 PMC8709944

[32] Shi Y Jin J Ji W Guan X. Therapeutic landscape in mutational triple negative breast cancer. Mol Cancer (2018) 17(1):99. doi: 10.1186/s12943-018-0850-9 30007403 PMC6046102

[33] Masuda Y Futamura M Kamino H Nakamura Y Kitamura N Ohnishi S. The potential role of Dfna5, a hearing impairment gene, in P53-mediated cellular response to DNA damage. J Hum Genet (2006) 51(8):652–64. doi: 10.1007/s10038-006-0004-6 16897187

[34] Zhang T Li Y Zhu R Song P Wei Y Liang T. Transcription factor P53 suppresses tumor growth by prompting pyroptosis in non-Small-Cell lung cancer. Oxid Med Cell Longev (2019) 2019:8746895. doi: 10.1155/2019/8746895 31737176 PMC6815571

[35] Hinshaw DC Shevde LA. The tumor microenvironment innately modulates cancer progression. Cancer Res (2019) 79(18):4557–66. doi: 10.1158/0008-5472.CAN-18-3962 PMC6744958 31350295

[36] Wu J Zhu Y Luo M Li L. Comprehensive analysis of pyroptosis-related genes and tumor microenvironment infiltration characterization in breast cancer. Front Immunol (2021) 12:748221. doi: 10.3389/fimmu.2021.748221 34659246 PMC8515898

[37] Stanton SE Adams S Disis ML. Variation in the incidence and magnitude of tumor-infiltrating lymphocytes in breast cancer subtypes: A systematic review. JAMA Oncol (2016) 2(10):1354–60. doi: 10.1001/jamaoncol.2016.1061 27355489

[38] Dieci MV Radosevic-Robin N Fineberg S van den Eynden G Ternes N Penault-Llorca F. Update on tumor-infiltrating lymphocytes (Tils) in breast cancer, including recommendations to assess tils in residual disease after neoadjuvant therapy and in carcinoma in situ: A report of the international immuno-oncology biomarker working group on breast cancer. Semin Cancer Biol (2018) 52(Pt 2):16–25. doi: 10.1016/j.semcancer.2017.10.003 29024776

[39] Yam C Yen EY Chang JT Bassett RL Alatrash G Garber H. Immune phenotype and response to neoadjuvant therapy in triple-negative breast cancer. Clin Cancer Res (2021) 27(19):5365–75. doi: 10.1158/1078-0432.CCR-21-0144 PMC8752638 34253579

[40] Adams S Gray RJ Demaria S Goldstein L Perez EA Shulman LN. Prognostic value of tumor-infiltrating lymphocytes in triple-negative breast cancers from two phase iii randomized adjuvant breast cancer trials: Ecog 2197 and ecog 1199. J Clin Oncol (2014) 32(27):2959–66. doi: 10.1200/JCO.2013.55.0491 PMC4162494 25071121

[41] Ali HR Provenzano E Dawson SJ Blows FM Liu B Shah M. Association between Cd8+ T-cell infiltration and breast cancer survival in 12,439 patients. Ann Oncol (2014) 25(8):1536–43. doi: 10.1093/annonc/mdu191 24915873

[42] Wu SY Xu Y Chen L Fan L Ma XY Zhao S. Combined angiogenesis and pd-1 inhibition for immunomodulatory tnbc: Concept exploration and biomarker analysis in the future-C-Plus trial. Mol Cancer (2022) 21(1):84. doi: 10.1186/s12943-022-01536-6 35337339 PMC8951705

[43] Noel G Fontsa ML Garaud S De Silva P de Wind A Van den Eynden GG. Functional Th1-oriented T follicular helper cells that infiltrate human breast cancer promote effective adaptive immunity. J Clin Invest (2021) 131(19):e139905. doi: 10.1172/JCI139905 34411002 PMC8483751

[44] Gu-Trantien C Loi S Garaud S Equeter C Libin M de Wind A. Cd4(+) follicular helper T cell infiltration predicts breast cancer survival. J Clin Invest (2013) 123(7):2873–92. doi: 10.1172/JCI67428 PMC3696556 23778140

[45] Siegers GM Dutta I Kang EY Huang J Kobel M Postovit LM. Aberrantly expressed embryonic protein nodal alters breast cancer cell susceptibility to gammadelta T cell cytotoxicity. Front Immunol (2020) 11:1287. doi: 10.3389/fimmu.2020.01287 32636849 PMC7319087

[46] Meraviglia S Eberl M Vermijlen D Todaro M Buccheri S Cicero G. *In vivo* manipulation of Vgamma9vdelta2 T cells with zoledronate and low-dose interleukin-2 for immunotherapy of advanced breast cancer patients. Clin Exp Immunol (2010) 161(2):290–7. doi: 10.1111/j.1365-2249.2010.04167.x PMC2909411 20491785

[47] Ju M Qi A Bi J Zhao L Jiang L Zhang Q. A five-mrna signature associated with post-translational modifications can better predict recurrence and survival in cervical cancer. J Cell Mol Med (2020) 24(11):6283–97. doi: 10.1111/jcmm.15270 PMC7294153 32306508

[48] Ning ZK Hu CG Huang C Liu J Zhou TC Zong Z. Molecular subtypes and Cd4(+) memory T cell-based signature associated with clinical outcomes in gastric cancer. Front Oncol (2020) 10:626912. doi: 10.3389/fonc.2020.626912 33816214 PMC8011500

[49] Liu D Vadgama J Wu Y. Basal-like breast cancer with low tgfbeta and high tnfalpha pathway activity is rich in activated memory Cd4 T cells and has a good prognosis. Int J Biol Sci (2021) 17(3):670–82. doi: 10.7150/ijbs.56128 PMC7975701 33767579

[50] Garaud S Buisseret L Solinas C Gu-Trantien C de Wind A Van den Eynden G. Tumor infiltrating b-cells signal functional humoral immune responses in breast cancer. JCI Insight (2019) 5:e129641. doi: 10.1172/jci.insight.129641 PMC6795287 31408436

[51] Gu-Trantien C Migliori E Buisseret L de Wind A Brohee S Garaud S. Cxcl13-producing tfh cells link immune suppression and adaptive memory in human breast cancer. JCI Insight (2017) 2(11):e91487. doi: 10.1172/jci.insight.91487 PMC5453706 28570278

[52] Hu Q Hong Y Qi P Lu G Mai X Xu S. Atlas of breast cancer infiltrated b-lymphocytes revealed by paired single-cell rna-sequencing and antigen receptor profiling. Nat Commun (2021) 12(1):2186. doi: 10.1038/s41467-021-22300-2 33846305 PMC8042001

[53] Helmink BA Reddy SM Gao J Zhang S Basar R Thakur R. B cells and tertiary lymphoid structures promote immunotherapy response. Nature (2020) 577(7791):549–55. doi: 10.1038/s41586-019-1922-8 PMC8762581 31942075

[54] Vito A Salem O El-Sayes N MacFawn IP Portillo AL Milne K. Immune checkpoint blockade in triple negative breast cancer influenced by b cells through myeloid-derived suppressor cells. Commun Biol (2021) 4(1):859. doi: 10.1038/s42003-021-02375-9 34253827 PMC8275624

[55] Tan Y Sun R Liu L Yang D Xiang Q Li L. Tumor suppressor Drd2 facilitates M1 macrophages and restricts nf-kappab signaling to trigger pyroptosis in breast cancer. Theranostics (2021) 11(11):5214–31. doi: 10.7150/thno.58322 PMC8039962 33859743

[56] Zhang Y Fan Y Jing X Zhao L Liu T Wang L. Otud5-mediated deubiquitination of yap in macrophage promotes M2 phenotype polarization and favors triple-negative breast cancer progression. Cancer Lett (2021) 504:104–15. doi: 10.1016/j.canlet.2021.02.003 33587979

[57] Mattiuz R Brousse C Ambrosini M Cancel JC Bessou G Mussard J. Type 1 conventional dendritic cells and interferons are required for spontaneous Cd4(+) and Cd8(+) T-cell protective responses to breast cancer. Clin Transl Immunol (2021) 10(7):e1305. doi: 10.1002/cti2.1305 PMC8279130 34277006

[58] Bates JP Derakhshandeh R Jones L Webb TJ. Mechanisms of immune evasion in breast cancer. BMC Cancer (2018) 18(1):556. doi: 10.1186/s12885-018-4441-3 29751789 PMC5948714

[59] Thorsson V Gibbs DL Brown SD Wolf D Bortone DS Ou Yang TH. The immune landscape of cancer. Immunity (2018) 48(4):812–30.e14. doi: 10.1016/j.immuni.2018.03.023 29628290 PMC5982584

[60] Luen S Virassamy B Savas P Salgado R Loi S. The genomic landscape of breast cancer and its interaction with host immunity. Breast (2016) 29:241–50. doi: 10.1016/j.breast.2016.07.015 27481651

[61] Stephens PJ Tarpey PS Davies H Van Loo P Greenman C Wedge DC. The landscape of cancer genes and mutational processes in breast cancer. Nature (2012) 486(7403):400–4. doi: 10.1038/nature11017 PMC3428862 22722201

[62] Tekpli X Lien T Rossevold AH Nebdal D Borgen E Ohnstad HO. An independent poor-prognosis subtype of breast cancer defined by a distinct tumor immune microenvironment. Nat Commun (2019) 10(1):5499. doi: 10.1038/s41467-019-13329-5 31796750 PMC6890706

[63] Denkert C von Minckwitz G Darb-Esfahani S Lederer B Heppner BI Weber KE. Tumour-infiltrating lymphocytes and prognosis in different subtypes of breast cancer: A pooled analysis of 3771 patients treated with neoadjuvant therapy. Lancet Oncol (2018) 19(1):40–50. doi: 10.1016/S1470-2045(17)30904-X 29233559

[64] Loi S Michiels S Salgado R Sirtaine N Jose V Fumagalli D. Tumor infiltrating lymphocytes are prognostic in triple negative breast cancer and predictive for trastuzumab benefit in early breast cancer: Results from the finher trial. Ann Oncol (2014) 25(8):1544–50. doi: 10.1093/annonc/mdu112 24608200

[65] Wang H Wu X Chen Y. Stromal-immune score-based gene signature: A prognosis stratification tool in gastric cancer. Front Oncol (2019) 9:1212. doi: 10.3389/fonc.2019.01212 31781506 PMC6861210

[66] Hou J Zhao R Xia W Chang CW You Y Hsu JM. Pd-L1-Mediated gasdermin c expression switches apoptosis to pyroptosis in cancer cells and facilitates tumour necrosis. Nat Cell Biol (2020) 22(10):1264–75. doi: 10.1038/s41556-020-0575-z PMC7653546 32929201

[67] Vonderheide RH LoRusso PM Khalil M Gartner EM Khaira D Soulieres D. Tremelimumab in combination with exemestane in patients with advanced breast cancer and treatment-associated modulation of inducible costimulator expression on patient T cells. Clin Cancer Res (2010) 16(13):3485–94. doi: 10.1158/1078-0432.CCR-10-0505 20479064

[68] Dongre A Rashidian M Eaton EN Reinhardt F Thiru P Zagorulya M. Direct and indirect regulators of epithelial-mesenchymal transition-mediated immunosuppression in breast carcinomas. Cancer Discovery (2021) 11(5):1286–305. doi: 10.1158/2159-8290.CD-20-0603 PMC8432413 33328216

[69] Popat S Hubner R Houlston RS. Systematic review of microsatellite instability and colorectal cancer prognosis. J Clin Oncol (2005) 23(3):609–18. doi: 10.1200/JCO.2005.01.086 15659508

[70] Deng H Zhao Y Cai X Chen H Cheng B Zhong R. Pd-L1 expression and tumor mutation burden as pathological response biomarkers of neoadjuvant immunotherapy for early-stage non-small cell lung cancer: A systematic review and meta-analysis. Crit Rev Oncol Hematol (2022) 170:103582. doi: 10.1016/j.critrevonc.2022.103582 35031441

[71] Ning B Liu Y Wang M Li Y Xu T Wei Y. The predictive value of tumor mutation burden on clinical efficacy of immune checkpoint inhibitors in melanoma: A systematic review and meta-analysis. Front Pharmacol (2022) 13:748674. doi: 10.3389/fphar.2022.748674 35355708 PMC8959431

[72] Baretti M Le DT. DNA mismatch repair in cancer. Pharmacol Ther (2018) 189:45–62. doi: 10.1016/j.pharmthera.2018.04.004 29669262

