# Supplementary material for: Immune landscape and risk prediction based on pyroptosis-related molecular subtypes in triple-negative breast cancer
Source: Front Immunol. 2022 Sep 16;13:933703. doi: 10.3389/fimmu.2022.933703 (PMC9524227; doi:10.3389/fimmu.2022.933703)
Supplement: Supplementary file 1 [file DataSheet_1.zip › SupplementaryFiguresTables20220830/Supplementary Figures 0830.docx]

**SUPPLEMENTARY FIGURES**


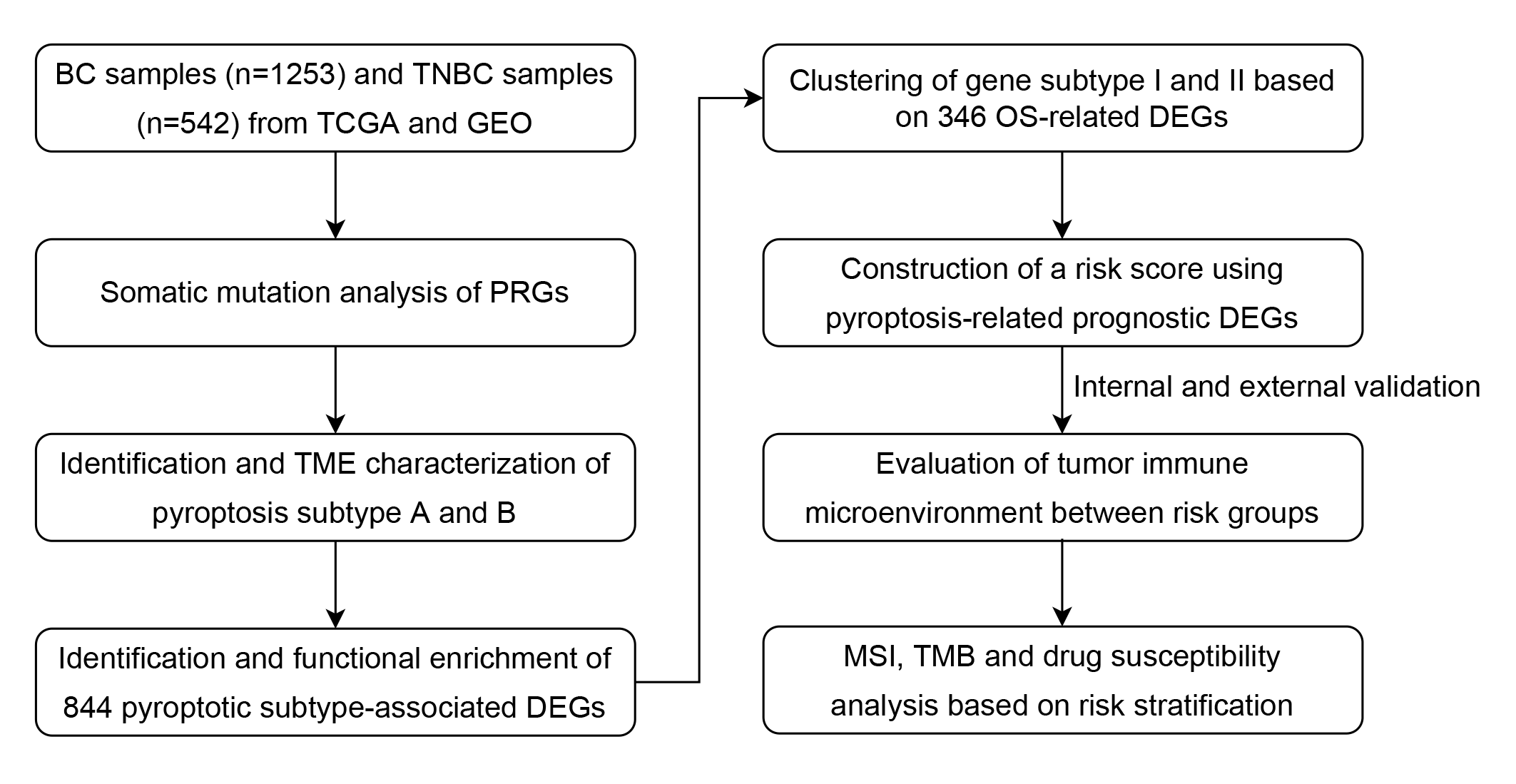


**Supplementary Figure S1.** Workflow of the present study.


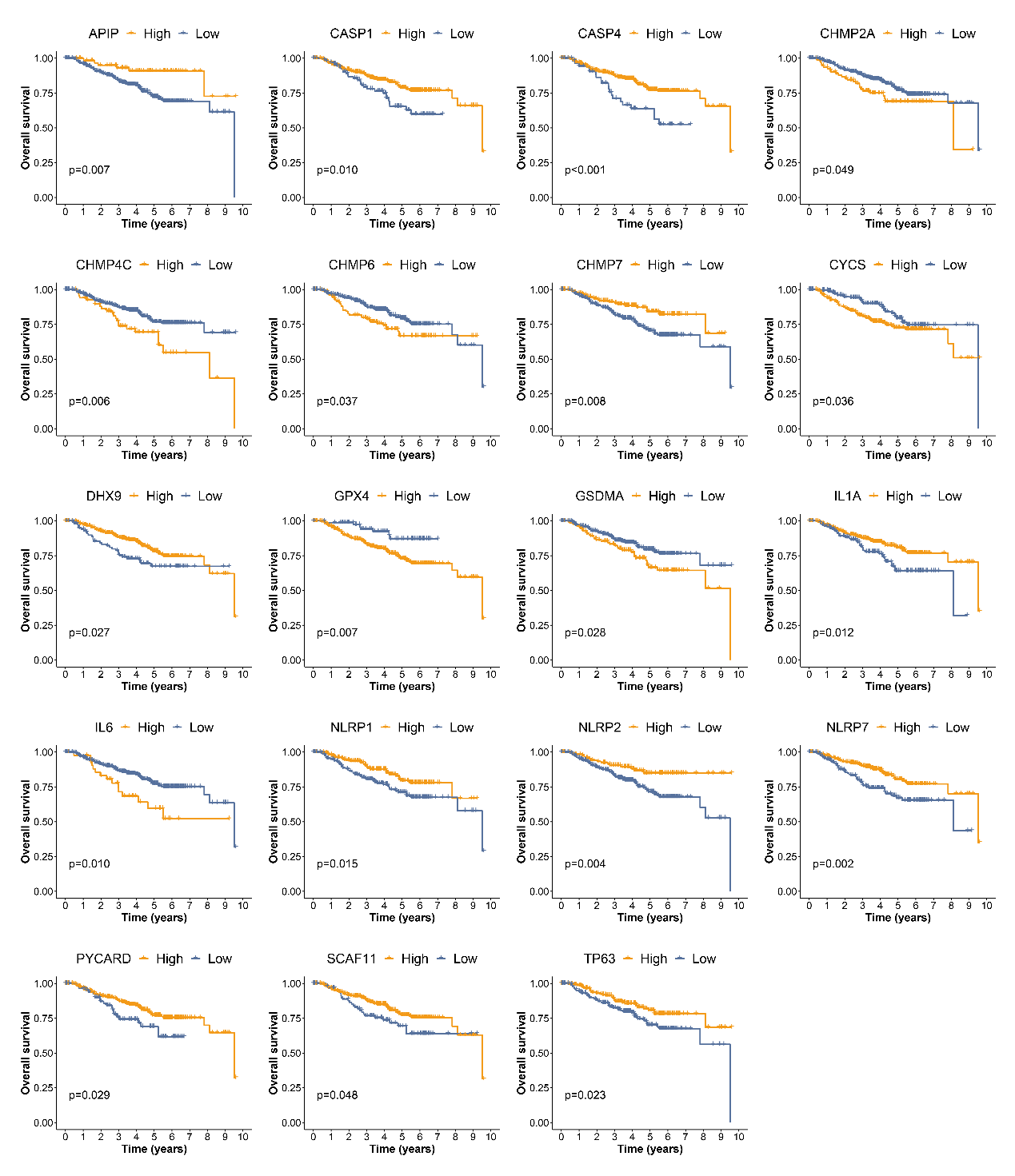


**Supplementary Figure S2.** Nineteen OS-related PRGs in TNBC in addition to the 12 PRGs in Figure 2.


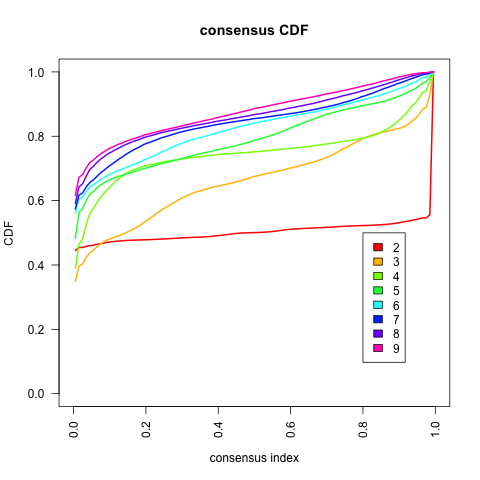


**Supplementary Figure S3.** Cumulative distribution function curves identified optimal k (k=2) for pyroptosis subtype clustering.


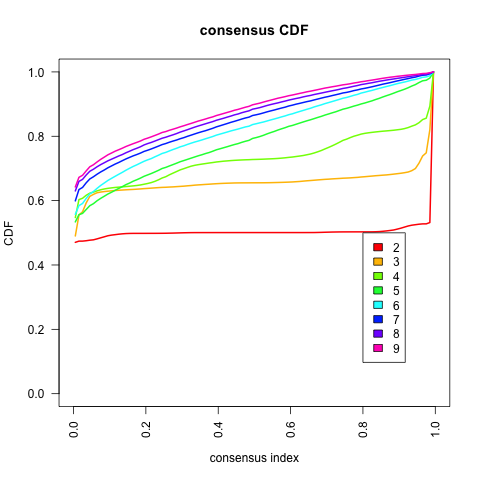


**Supplementary Figure S4.** Cumulative distribution function curves identified optimal k (k=2) for gene subtype clustering.


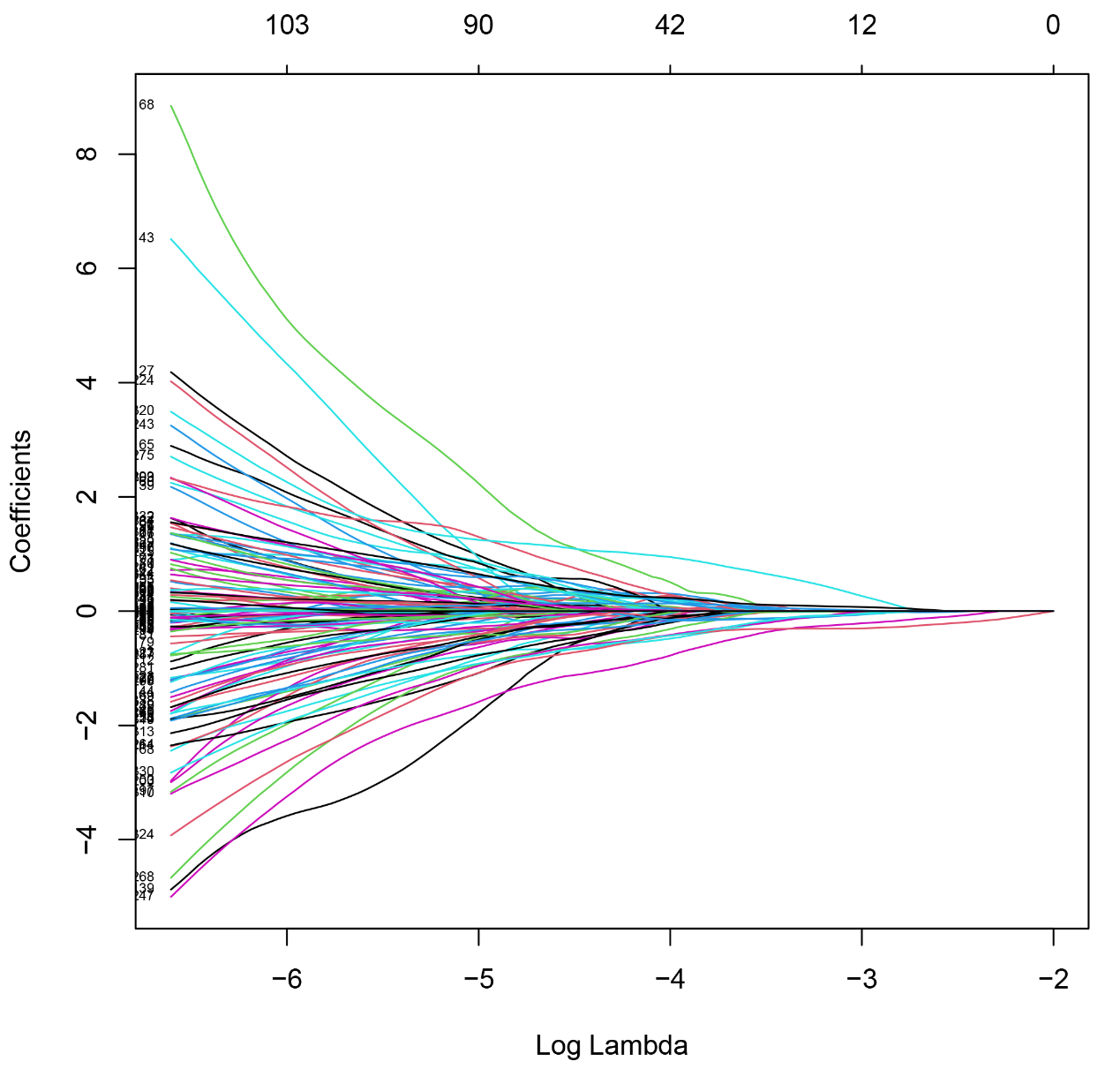


**Supplementary Figure S5.** A 10-fold cross-validation for parameter selection was performed using the Lasso model. Two dotted vertical lines were drawn using the minimum criteria (the value of lambda that gives a minimum mean cross-validated error) and one standard error away from the minimum criteria.


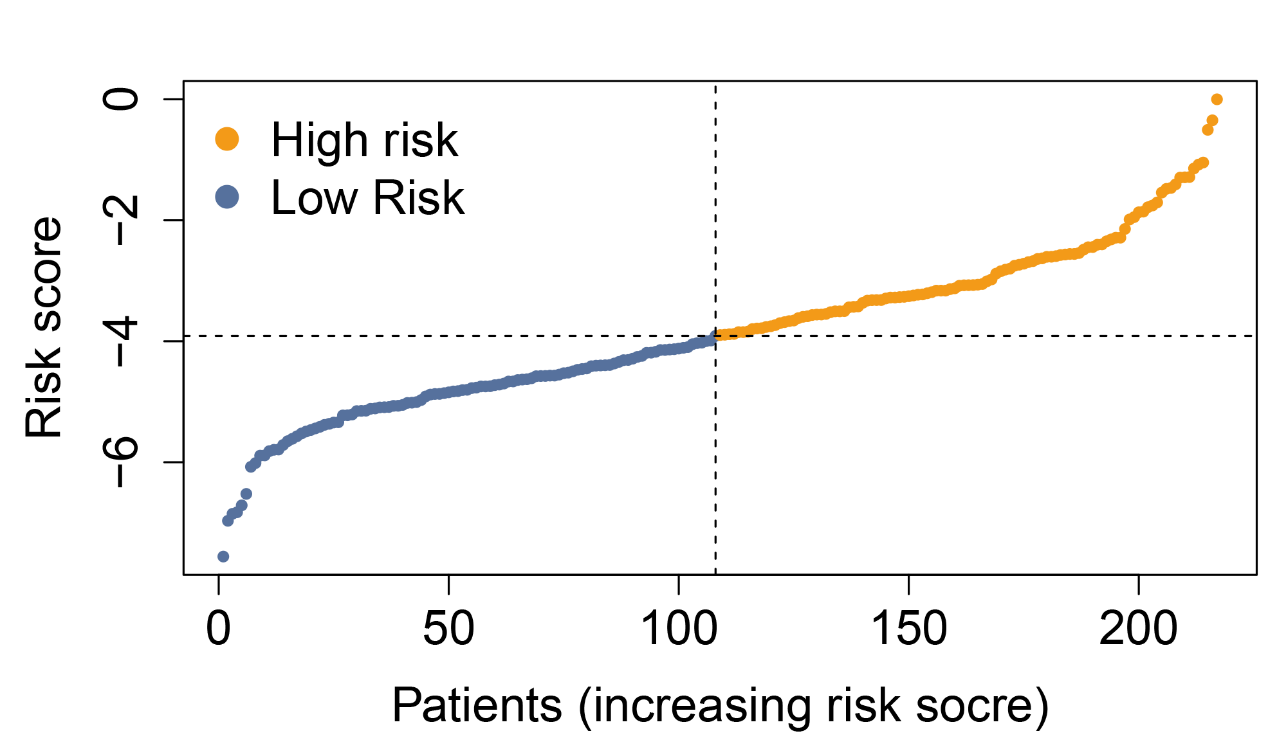


**Supplementary Figure S6.** Risk score distribution in patients from internal validation set.


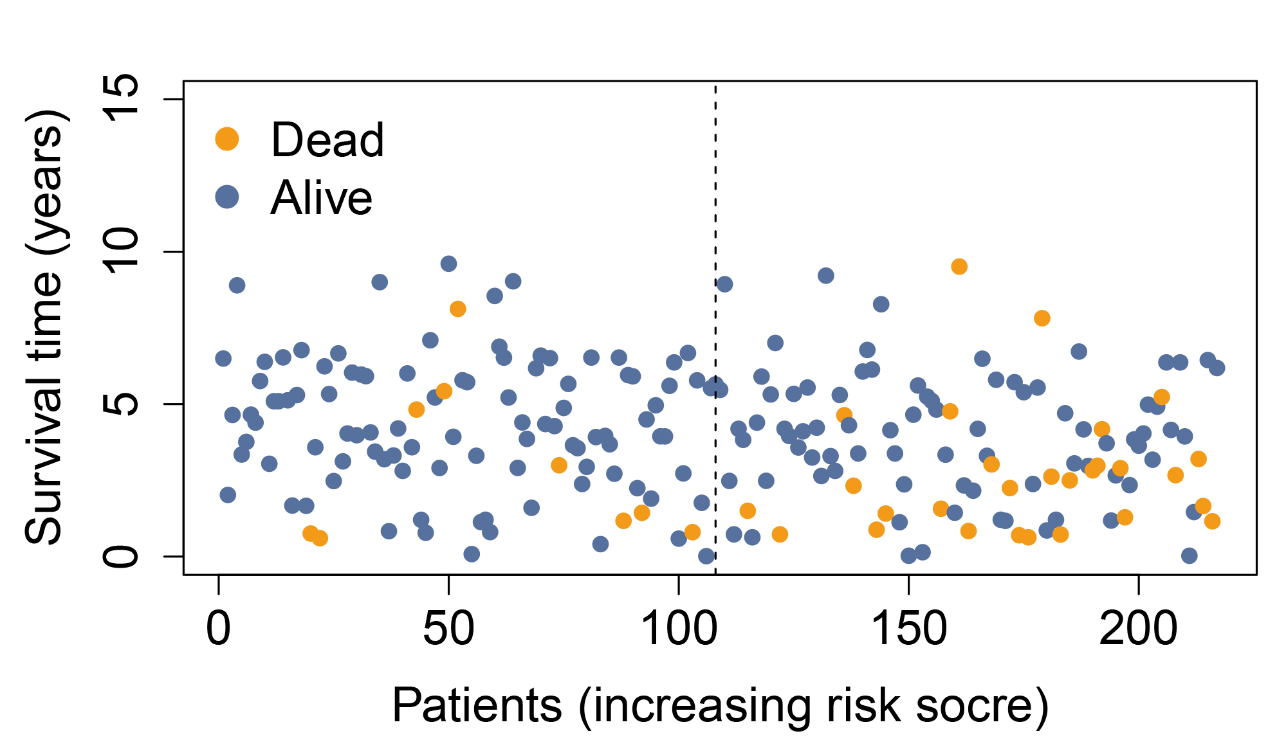


**Supplementary Figure S7.** Vital status plot showed higher death rate in patients from high-risk group in internal validation set.


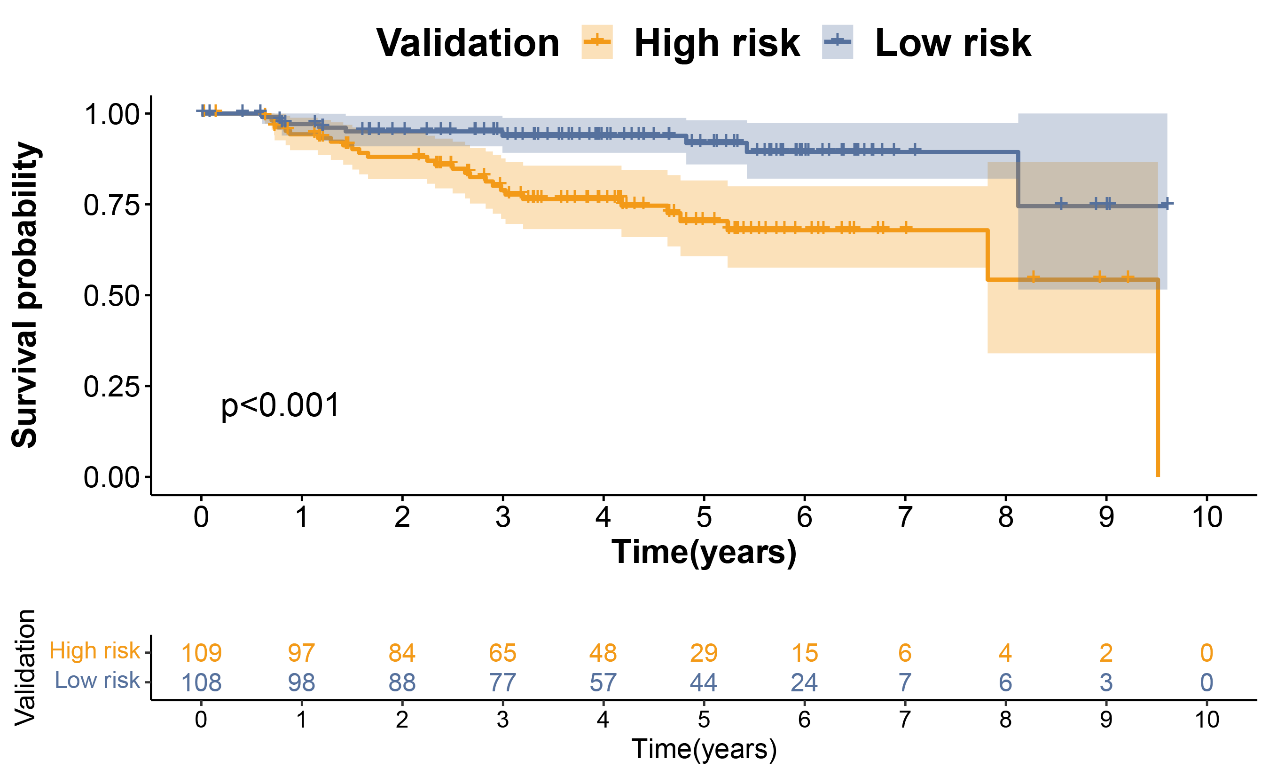


**Supplementary Figure S8.** Remarkably better OS was observed in low-risk group in internal validation set.


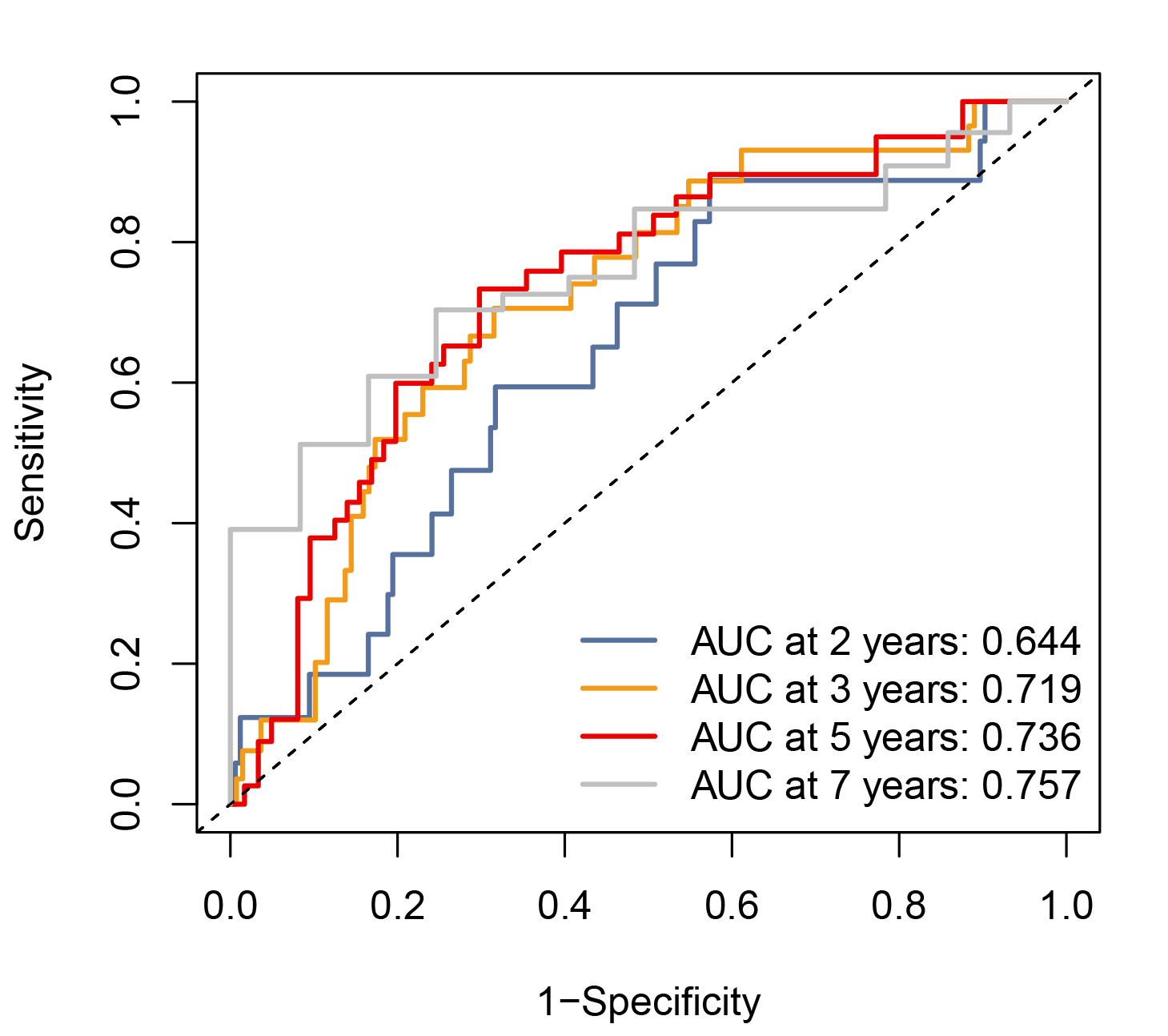


**Supplementary Figure S9.** ROC curves predicted the sensitivity and specificity of the 12-gene-based risk score in predicting 2-, 3-, 5- and 7-year OS for patients in internal validation set.


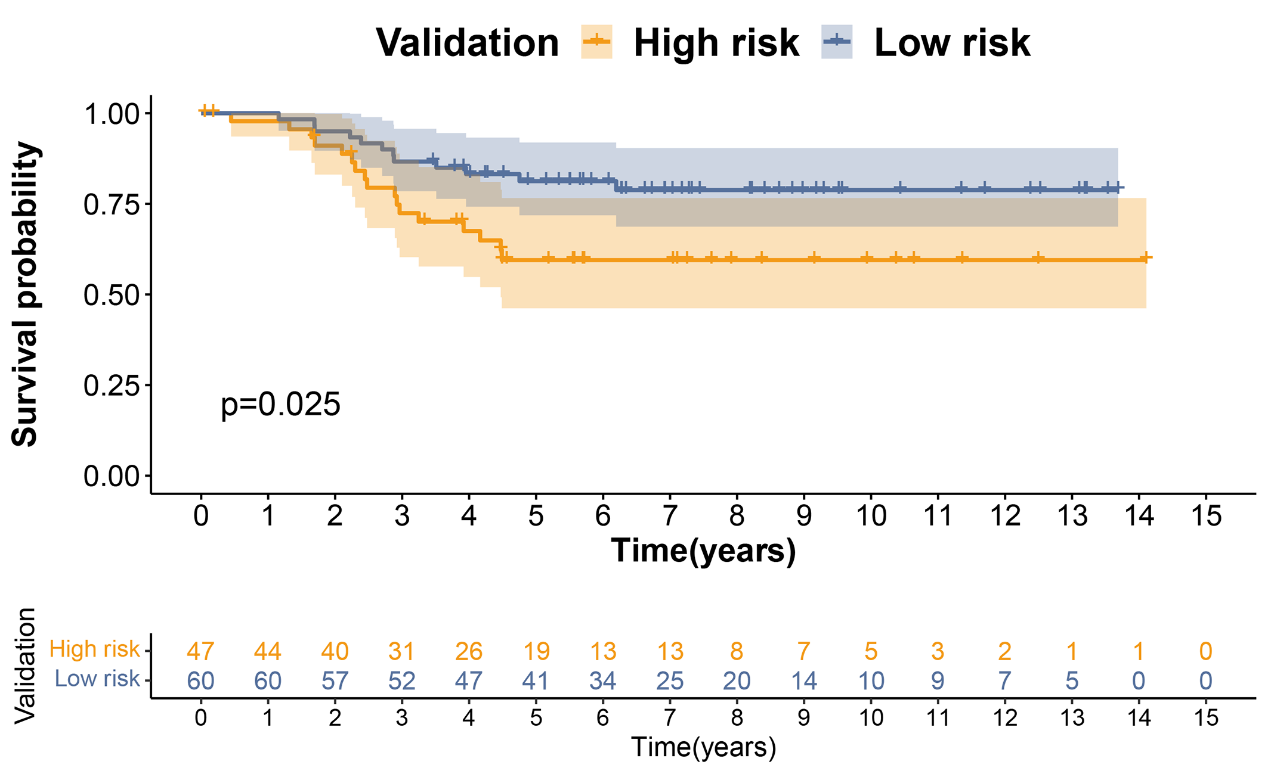


**Supplementary Figure S10.** Better long-term OS of low-risk patients was verified in external validation cohort (GSE58812).


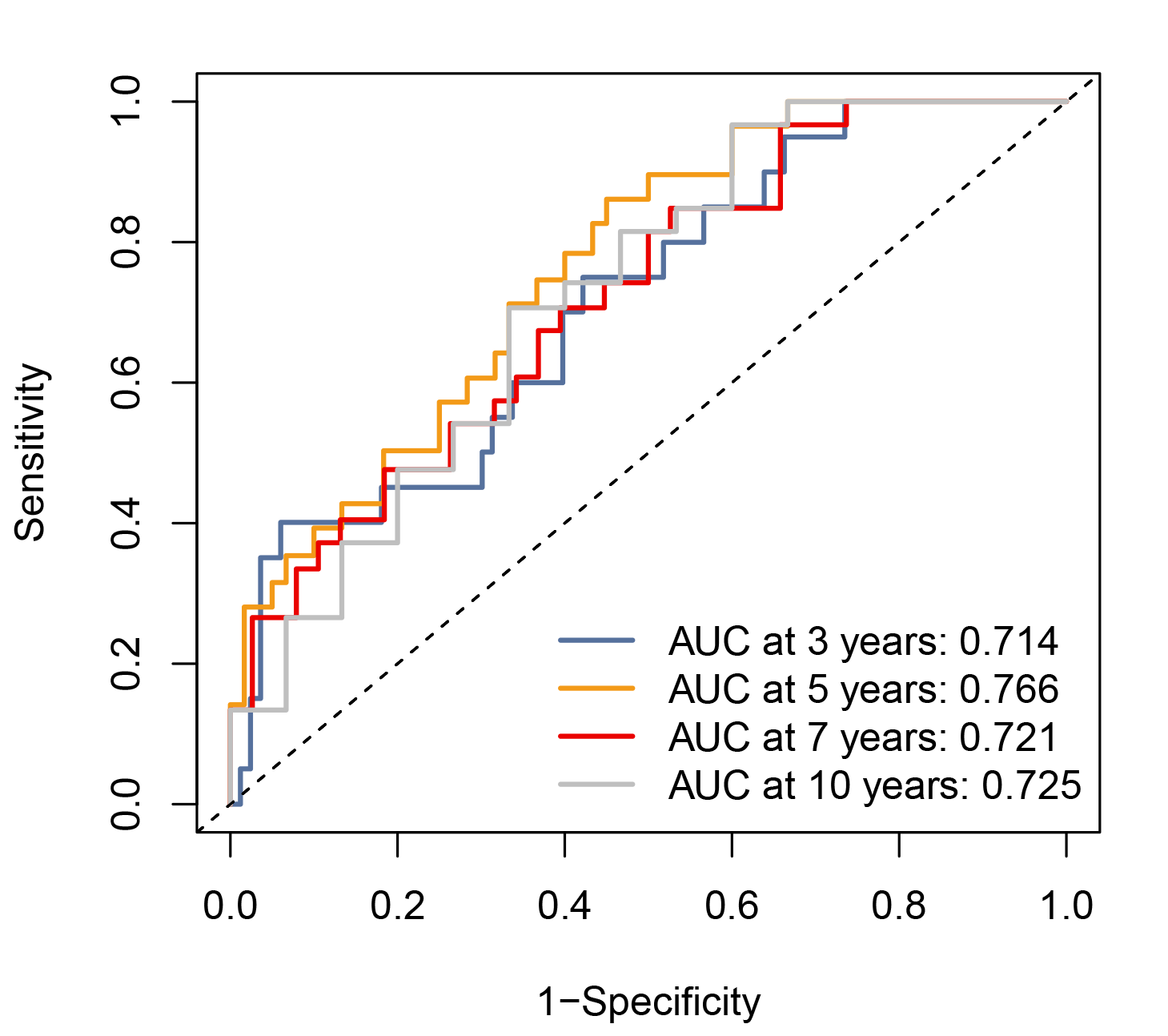


**Supplementary Figure S11.** ROC curves performed in external validation cohort (GSE58812) demonstrated a good performance of the 12-DEG-based risk score in prediction of long-term prognosis for TNBC patients.


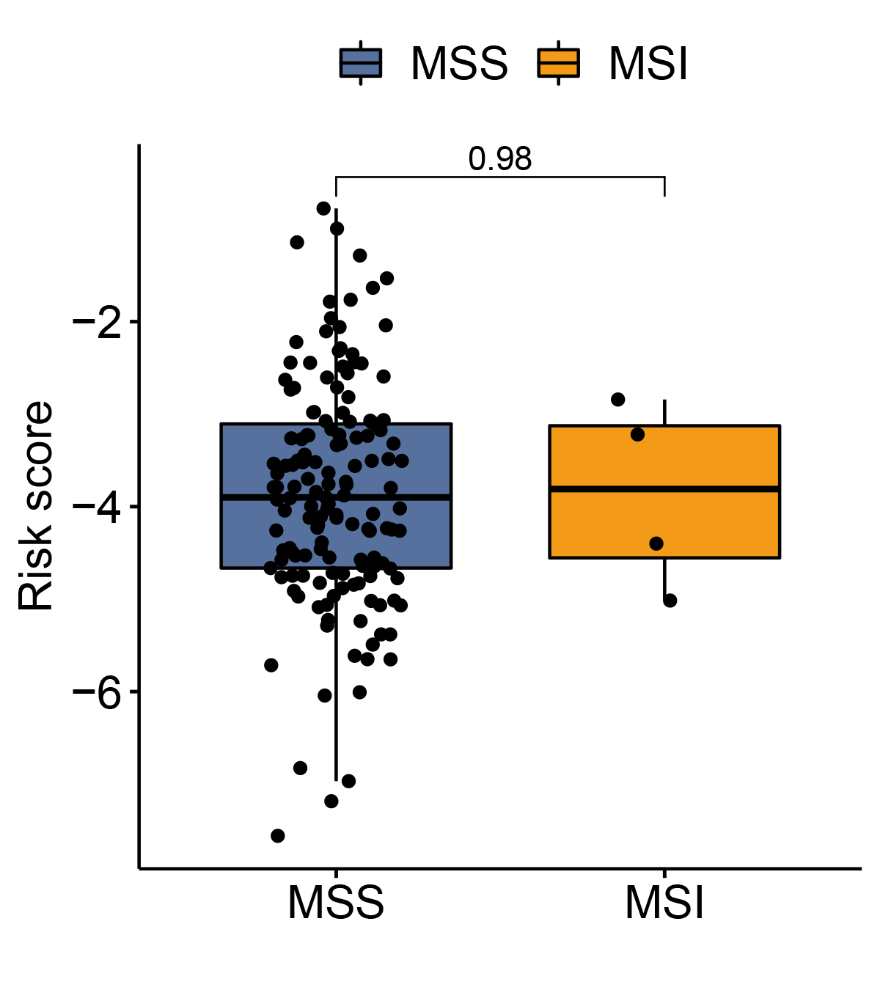


**Supplementary Figure S12.** No difference in risk score distribution was observed between MSI and MSS cases.


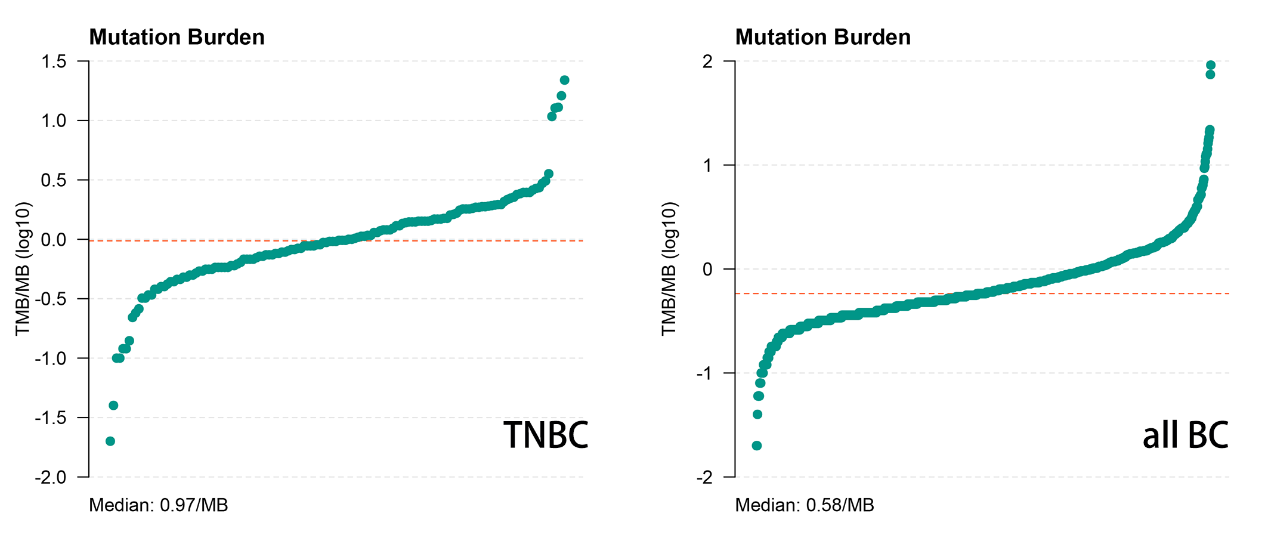


**Supplementary Figure S13.** Tumor mutation burden of TNBC and all BC samples.
